# eEF2K Activity Determines Synergy to Cotreatment of Cancer Cells With PI3K and MEK Inhibitors

**DOI:** 10.1016/j.mcpro.2022.100240

**Published:** 2022-05-02

**Authors:** Maruan Hijazi, Pedro Casado, Nosheen Akhtar, Saul Alvarez-Teijeiro, Vinothini Rajeeve, Pedro R. Cutillas

**Affiliations:** 1 Signalling & Proteomics Group, Centre for Genomics and Computational Biology, Barts Cancer Institute, Queen Mary University of London, London, United Kingdom; 2 The Alan Turing Institute, British Library, London, United Kingdom

**Keywords:** phosphoproteomics, kinase activity, eEF2K, cancer, AML, biomarkers, synergy, combination therapy, 4EBP1, eukaryotic translation initiation factor 4E-binding protein 1, ACN, acetonitrile, AML, acute myeloid leukemia, CDI, coefficient of drug interaction, DMSO, dimethyl sulfoxide, eEF2, eukaryotic elongation factor 2, eEF2K, eukaryotic elongation factor 2 kinase, ERK, extracellular signal-related kinase, FBS, fetal bovine serum, FDA, Food and Drug Administration, HEL, human erythroleukemia, IRS1, insulin receptor substrate 1, JNK1, c-Jun N-terminal kinase 1, LDS, lithium dodecyl sulfate, MCF7, Michigan Cancer Foundation-7, MEK, MAPK/ERK kinase, MAPK, mitogen-activated protein kinase, MOPS, 3-(N-morpholino) propanesulfonic acid, MS/MS, tandem mass spectrometry, mTOR, mammalian target of rapamycin, NaF, sodium fluoride, PRAS40, proline-rich AKT1 substrate 1, RIPA, radioimmunoprecipitation assay, THP-1, Tohoku Hospital Pediatrics-1, TiO2, titanium dioxide, XIC, extracted ion chromatogram

## Abstract

PI3K-mammalian target of rapamycin and MAPK/ERK kinase (MEK)/mitogen-activated protein kinase (MAPK) are the most frequently dysregulated signaling pathways in cancer. A problem that limits the success of therapies that target individual PI3K-MAPK members is that these pathways converge to regulate downstream functions and often compensate each other, leading to drug resistance and transient responses to therapy. In order to overcome resistance, therapies based on cotreatments with PI3K/AKT and MEK/MAPK inhibitors are now being investigated in clinical trials, but the mechanisms of sensitivity to cotreatment are not fully understood. Using LC-MS/MS-based phosphoproteomics, we found that eukaryotic elongation factor 2 kinase (eEF2K), a key convergence point downstream of MAPK and PI3K pathways, mediates synergism to cotreatment with trametinib plus pictilisib (which target MEK1/2 and PI3Kα/δ, respectively). Inhibition of eEF2K by siRNA or with a small molecule inhibitor reversed the antiproliferative effects of the cotreatment with PI3K plus MEK inhibitors in a cell model–specific manner. Systematic analysis in 12 acute myeloid leukemia cell lines revealed that eEF2K activity was increased in cells for which PI3K plus MEKi cotreatment is synergistic, while PKC potentially mediated resistance to such cotreatment. Together, our study uncovers eEF2K activity as a key mediator of responses to PI3Ki plus MEKi and as a potential biomarker to predict synergy to cotreatment in cancer cells.

## Highlights

• eEF2K mediates synergy to cotreatment with PI3K and MAPK/ERK kinase (MEK) inhibitors
• eEF2K silencing rescued the antiproliferative effects of PI3Ki + MEKi
• Basal eEF2K activity predicts synergy to PI3Ki + MEKi cotreatment in leukemia

## Graphical Abstract

**Figure:**
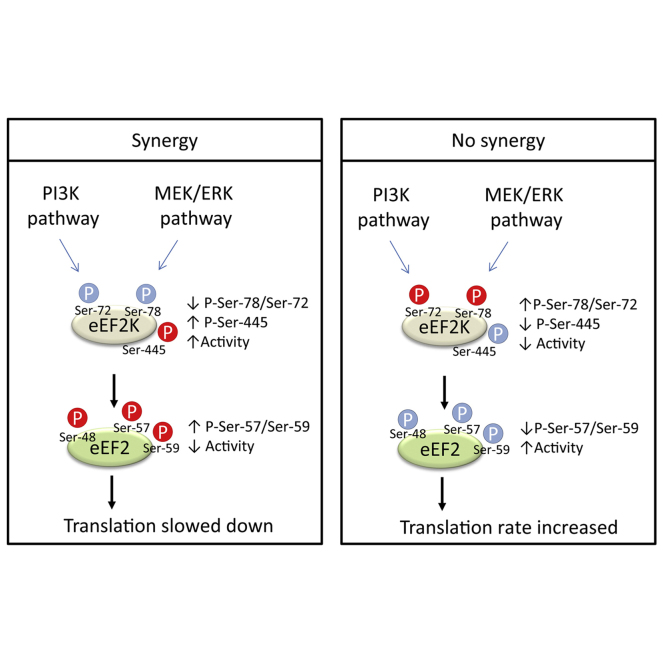

## In Brief

Using a phosphoproteomic approach, we found that eEF2K mediates synergy to treatment with trametinib plus pictilisib (MEK and PI3K inhibitors, respectively). An eEF2K activity signature was highly associated with sensitivity to cotreatment in a panel of 12 AML cell lines. Thus, our study uncovers eEF2K as a key mediator of responses to PI3Ki plus MEKi and a protein activity signature that predicts synergistic response to cotreatments with MEK plus PI3K inhibitors.

Mitogen-activated protein kinase (MAPK) and PI3K-mammalian target of rapamycin (mTOR) signaling pathways play key functions in the regulation of cell survival, differentiation, proliferation, metabolism, and motility in response to extracellular stimuli and are deregulated in most cancers (1). Consequently, pharmacological inhibitors of the MAPK-PI3K network are intensively pursued therapeutic targets for the treatment of different cancer types.

Kinase-targeted therapies are effective in tumors addicted to prosurvival signals from the target. Such oncogenic addiction may be caused by activating mutations, target overexpression, or signals from upstream pathways. However, in addition to activation of the kinase target, responses to kinase inhibitors are also determined by the activation of pathways with the potential to compensate for target inhibition (2). Since signaling circuits are cell type- and patient-specific, cancer cells, even those of the same tumor type, show differences in signaling network wirings (3, 4), which in turn determine intrinsic therapeutic resistance to single oncogenic pathway inhibition (5). The problem of intrinsic resistance may be illustrated with alpelisib, a specific inhibitor of PI3Kα isoform (encoded by the *PIK3CA* gene); a recent clinical trial has shown that this drug produced a response rate of just 26.6% in the eligible patient population (*PIK3CA* mutant and hormone receptor-positive breast cancer patients) (6).

A common mechanism of resistance to PI3K inhibition involves the activation of kinase pathways, such as those driven by MAPK and PKC signaling enzymes, which compensate for target inhibition by acting in parallel to PI3K (7, 8). To overcome resistance, co-inhibition of PI3K-mTOR and MAPK pathways has been successful in reducing tumor growth in xenograft cancer models and in genetically engineered mice (9, 10). Consequently, clinical trials are evaluating combination therapies with inhibitors against PI3K and MAPK signaling members, such as PI3K, AKT, mTOR, BRAF, and MEK, which are approved to treat different cancers as monotherapies or in combination with other agents (11, 12, 13). However, the mechanisms that mediate synthetic lethality of PI3K and MAPK pathway inhibitors in some but not all tumors are not well understood, and this precludes selecting the most appropriate patient cohorts for testing cotreatment regimes.

Here, using a phosphoproteomics screen, we found that eEF2K, a kinase involved in the regulation of translation elongation, mediates sensitivity to cotreatment with PI3K and MAPK pathway inhibitors in different cancer types. Consistent with this notion, genetic and pharmacological eEF2K inhibition reversed the antiproliferative effects of PI3K-MAPK pathway inhibitors. In addition, a signature of eEF2K activity (as measured by its autophosphorylation and phosphorylation of its substrate eEF2) was found to be associated with sensitivity to cotreatment in cell models of acute myeloid leukemia (AML) and could thus represent a biomarker signature to predict synergistic response to cotreatments with MEK and PI3K inhibitors.

## Experimental Procedures

### Cell Culture

The cell lines NTERA-2, CMK, KASUMI-1, human erythroleukemia (HEL), ML-2, MOLM-13, MV4-11, OCI-AML2, P31/FUJ, and THP-1 were derived from male patients, while MCF-7, KMOE-2, HL60, and NOMO-1 were derived from female patients.

MCF7 and NTERA2 cells were routinely cultured using Dulbecco’s modified Eagle’s medium (10% fetal bovine serum [FBS], 1% penicillin/streptomycin) at 37 °C, in a humidified atmosphere containing 5% CO_2_. The culture medium for all AML cell lines was RPMI (supplemented with 10% FBS, 1% penicillin/streptomycin), except OCI-AML2 that required α-minimum essential medium for growing (supplemented with 20% FBS). Cell density was kept between 0.1 and 0.5 × 10^6^ cells per mL. Each kinase inhibitor was diluted to 1000 times the desired concentration for treatment using dimethyl sulfoxide (DMSO). Each compound was then added to the cell population at a 1:1000 dilution in the culture medium. Depending on the type of experiment, cells were incubated at different time points, and compounds were added at various concentrations prior to lysis. Trametinib (MEK inhibitor) and GDC-0941 (PI3K inhibitor) were purchased from Selleckchem (S2673 and S1065, respectively), and A484954 (eEF2Ki) was purchased from Tocris (Cat nº 4483). The eEF2Ki was used at a concentration of 10 μM for NTERA2 and MCF7 and 50 μM for HL60 cells.

### Cell Viability and Survival Assays

AML cell lines were directly seeded in 96-well plates (3 × 10^4^ cells per well in 150 μl) and treated with vehicle (DMSO) or 10 to 1000 nM of the indicated inhibitor for 72 h. Final concentration of DMSO was kept at 0.1%. Trypsin was used for cell dissociation from adherent cell lines (MCF7 and NTERA2). Cells were stained with Guava ViaCount reagent (Merck Millipore, cat. 4000–0040) according to the manufacturer’s instructions. Cell viability and survival were measured with a Guava PCA cell analyzer (Guava Technologies Inc) to generate flow cytometry data, which were analyzed using CytoSoft (v2.5.7). Kinase inhibitors treatments in NTERA2, MCF7, and HL60 were performed in four technical replicates and three biological replicates. For the rest of AML cell lines, measurements were performed in three technical replicates. Viability and survival values were averaged and expressed relative to vehicle control.

### siRNA Transfection

Cells were transfected with a validated nontargeting siRNA (NT-siRNA) or with ON-TARGETplus SMART pool against human *eEF2K* siRNA (L-004950–00–0005) obtained from Dharmacon, Inc. Transfections were performed with Lipofectamine 3000 reagent, according to the manufacturer’s instructions. Cell treatments were performed 72 h after transfections. After the siRNA transfection, the reduction in protein expression was assessed by Western blot analysis.

### Immunoblotting

MCF7 and NTERA2 cells were seeded in 6-well plates at a density of 0.3 to 0.4 × 10^6^ cells per well. The following day, cells were treated with kinase inhibitors for different time points and then washed twice with ice-cold PBS (supplemented with 1 mM Na_3_VO_4_ and 1 mM sodium fluoride [NaF]). Radioimmunoprecipitation assay (RIPA) buffer (150 μl) (50 mM Tris-HCl, pH 7.6, 150 mM NaCl, 1 mM EDTA, 1% NP-40, 0.5% sodium deoxycholate, 0.1% SDS) supplemented with 1 mM Na_3_VO_4_, 1 mM NaF, 1X protease inhibitor cocktail, 1 mM PMSF, and 0.5 μM okadaic acid was added to each well. Cells were scraped, and the resulting lysate collected. For AML cell lines, cells were seeded in T25 flasks at a density of 0.5-1x10^6^ cells/ml. After treatment, cells were collected by centrifugation, washed twice with ice-cold PBS (supplemented with 1 mM Na_3_VO_4_ and 1 mM NaF). Finally, pellets were resuspended in 200 μl of supplemented RIPA buffer. Cell lysates were sonicated for 10 min (10 cycles of 30 s on 35 s off; Diagenode Bioruptor Plus) and centrifuged at 16,000*g* for 15 min at 4 °C. Protein in the cell extracts was quantified by bicinchoninic acid analysis. 30 to 60 μg (MCF7 and NTERA2), and 100 to 125 μg (AML cell lines) of protein extract were analyzed in 4 to 12% precast commercial gels (NuPAGE Novex 4–12% Bis-Tris Midi Gel 1.0 mm). The buffer used for protein electrophoresis was NuPAGE MOPS SDS running buffer 20x. 10 mM DTT and NuPAGE LDS sample buffer 4x were used to prepare the samples. Electrophoresis was run at room temperature using a constant voltage. After electrophoresis, gels were washed in transfer buffer (10% methanol, 0.1% NuPAGE Antioxidant diluted in NuPAGE transfer buffer 20x) for 10 min. Then, the proteins separated were transferred to a nitrocellulose membrane (iBlot Gel Transfer Stacks Nitrocellulose) for 13 min, applying a constant voltage. After blocking for nonspecific binding, the membranes were incubated for 14 h at 4 °C with primary antibody. Antibodies against GAPDH or β-Actin were used to quantify and normalize protein expression. Following this, after several washes, membranes were incubated with secondary antibody against mouse or rabbit immunoglobulin conjugated with peroxidase. Finally, membranes were incubated for 1 min with 1X SuperSignal West Pico ECL solution, which afforded a chemiluminescence reaction. Antibody affinity was then visualized using a ChemiDoc system, and the bands were quantified using Image Studio Lite (v5.2).

### Puromycin Incorporation Assay

Cell lines were cultured overnight at a density of 0.3-1x10^6^ cells per mL and the following day incubated with the MEKi and PI3Ki individually or in combination at 500 nM for 5 h. Then, 2 μM puromycin (Sigma-Aldrich 540,411) was added into the cultures for further 25 min. Samples were collected in RIPA buffer as follows above in the immunoblotting section. Antipuromycin antibody (Merck Millipore MABE343) was used to detect puromycin incorporated into proteins.

### Sample Preparation for Proteomics and Phosphoproteomics Analysis

After cell counting, MCF7 and NTERA2 cells were seeded in 100 cm^2^ Petri dish (0.25 × 10^6^ and 0.45 × 10^6^ cells/ml, respectively) and maintained in an incubator overnight at 37 °C and 5% CO_2_. Cells were washed twice with ice-cold PBS (supplemented with 1 mM Na_3_VO_4_ and 1 mM NaF) and lysed in 500 μl urea buffer (8 M urea in 20 mM in Hepes pH 8.0 supplemented with 1 mM Na_3_VO_4_, 1 mM NaF, 1 mM Na_4_P_2_O_7_, and 1 mM sodium β-glycerophosphate). Cell lysates were placed in low binding tubes, snap frozen, and store at −80 °C until further processing. For AML cell lines, 10 ml of cell suspension were seeded in T25 flasks (0.5-1x10^6^ cells/ml) and maintained in an incubator overnight at 37 °C and 5% CO_2_. Cells were harvested by centrifugation at 500*g* at 4 °C for 5 min, washed twice with ice-cold PBS supplemented with 1 mM Na_3_VO_4_ and 1 mM NaF, snap frozen, and stored at −80 °C until further processing. Cell pellets were lysed in urea buffer for 30 min. All cell lysates were homogenized by sonication, and insoluble material was removed by centrifugation at 16,000*g* for 15 min at 4 °C. Protein in the cell extracts was quantified by BCA analysis.

For phosphoproteome analysis, we used published methods (14, 15, 16) with some modifications. Briefly, 350 μg of protein were reduced and alkylated by sequential incubation with 10 mM DTT and 17 mM iodoacetamide for 1 h. The urea concentration was diluted to 2 M with 20 mM Hepes (pH 8.0) and 100 μl of conditioned trypsin beads (50% slurry of tosyl lysyl chloromethyl ketone–trypsin) (Thermo Fisher Scientific; Cat. 20230) were added, and the samples incubated for 16 h at 37 °C with agitation. Trypsin beads were removed by centrifugation at 2000*g* for 5 min at 4 °C. For proteomics analysis, 50 μg of protein were used.

Following trypsin digestion, peptide solutions were desalted using 10 mg OASIS-HLB cartridges (Waters). Briefly, OASIS cartridges were accommodated in a vacuum manifold (-5 mmHg), activated with 1 ml acetonitrile (ACN) and equilibrated with 1.5 ml washing solution (1% ACN, 0.1% TFA). After loading the samples, cartridges were washed twice with 0.75 ml of washing solution. For phosphoproteomics analysis, peptides were eluted with 500 μl of glycolic acid buffer 1 (1 M glycolic acid, 50% ACN, 5% TFA) and subjected to phosphoenrichment. For proteomics analysis, peptides were desalted using C18 spin tips and eluted with 500 μl of ACN solution (70% ACN, 0.1% TFA), dried in a speedvac (RVC 2–25, Martin Christ Gefriertrocknungsanlagen GmbH), and stored at −80 °C.

Phosphopeptides were enriched using TiO_2_ (GL Sciences) as previously described with some modifications (17, 18). Sample volumes were normalized to 0.5 ml using glycolic acid buffer 2 (1 M glycolic acid, 80% ACN, 5% TFA), and 25 μl of TiO_2_ beads (50% slurry in 1% TFA) were added to the peptide mixture, incubated for 5 min at room temperature with agitation, and centrifuged for 30 s at 1500*g*. For each sample, 80% of the supernatant was transferred to fresh tubes and stored in ice, and the remaining 20% used to resuspend the bead pellets that were loaded into an empty prewashed PE-filtered spin-tips (GlyGen) and packed by centrifugation at 1500*g* for 3 min. After loading the remaining volume of the supernatant by centrifugation at 1500*g* for 3 min, spin tips were sequentially washed with 100 μl of glycolic acid buffer 2, ammonium acetate buffer (100 mM ammonium acetate in 25% ACN), and 10% ACN by centrifugation for 3 min at 1500*g*. For phosphopeptide recovery, the addition of 50 μl of 5% ammonium water followed by centrifugation for 3 min at 1500*g* was repeated four times. Eluents were snap frozen in dry ice and dried in a speed vac, and phosphopeptide pellets stored at −80 °C.

### Mass Spectrometry

Phosphopeptide pellets were resuspended in 18 μl of reconstitution buffer (20 fmol/μl enolase in 3% ACN, 0.1% TFA), and 5 μl were loaded onto an LC-MS/MS system consisting of a Dionex UltiMate 3000 RSLC directly coupled to an Orbitrap Q-Exactive Plus mass spectrometer (Thermo Fisher Scientific). For proteomics, pellets were resuspended in reconstitution buffer (0.5 μg/μl), and 2 μl were injected. The LC system used mobile phases A (3% ACN; 0.1% formic acid [FA]) and B (100% ACN; 0.1% FA). Peptides were trap in a μ-precolumn (cat. 160454) and separated in an analytical column (Acclaim PepMap 100, cat. 164569). The following parameters were used: 3% to 23% B gradient for 60 min (phosphoproteomics) and 120 min (proteomics) and a flow rate of 0.3 μl/min.

As they eluted from the nano-LC system, peptides were infused into the online connected Q-Exactive Plus system operating with a 2.1 s duty cycle. Acquisition of full-scan survey spectra (m/z 375–1500) with a 70,000 full-width half-maximum resolution was followed by data-dependent acquisition in which the 15 most intense ions were selected for higher energy collisional dissociation and MS/MS scanning (200–2000 m/z) with a resolution of 17,500 full-width half-maximum. A 30 s dynamic exclusion period was enabled with an exclusion list with 10 ppm mass window. Overall duty cycle generated chromatographic peaks of approximately 30 s at the base, which allowed the construction of extracted ion chromatograms (XICs) with at least 10 data points.

### Peptide Identification From Tandem Mass Spectrometry Data

Mascot Daemon 2.5.0 was used to automate peptide identification from MS data. Peak list files (MGFs) from RAW data were generated with Mascot Distiller v2.5.1.0 and loaded into the Mascot search engine (v2.5) in order to match MS/MS data to peptides (19). Supplementary Dataset one included RAW file names linked to mzID files. The searches were performed against the SwissProt Database (SwissProt_Sep2014_2015_12.fasta) against “*Homo sapiens*”, and the number of entries used for the searches was 20,194 sequences, with a false discovery rate of ∼1% and the following parameters: two trypsin missed cleavages, mass tolerance of ±10 ppm for the MS scans and ±25 mmu for the MS/MS scans, carbamidomethyl Cys as a fixed modification, pyroGlu on N-terminal Gln, and oxidation of Met as variable modifications. Mascot calculated false discovery rate by comparing results against a decoy database. For phosphoproteomics experiments phosphorylation on Ser, Thr and Tyr was also included as variable modifications.

### Peptide Quantification From MS1 Data

The in-house developed Pescal software was used for label-free peptide quantification (20). Peptides and phosphopeptides were quantified using a previously described (7, 18, 21) label-free method that uses XIC. Briefly, XICs for all identified peptides across all samples were constructed with ± 7 ppm and ± 2 min mass and retention time windows, respectively. Then, peptide intensity values were determined as the calculated peak areas of the constructed XICs. Peptide intensities for each sample were normalized to the sum of all peptide intensity values in the same sample.

### Bioinformatics

Network analysis and visualization were performed using Gephi (version 0.9.2). The network was deployed using the Force Atlas two algorithm, making the graph visually readable and similar to many subnetworks. A community detection algorithm called modularity was highlighted, which depends on the comparison of the densities of edges within a group and from the group toward the rest of the network.

### Experimental Design and Statistical Rationale

The number of technical replicates and independent experiments is indicated for each experiment in its corresponding figure legend. Statistical analysis was performed in RStudio (v1.2.5033) or GraphPad Prism 8. Two-way ANOVA and further Bonferronis’s or Dunnett’s multiple comparisons tests were used to assess significance in Western blot, cell viability, and cell survival data. Pearson r was calculated in correlation analysis, and Wilcoxon signed-rank test was used to compare two related groups. Unpaired, two-tail Student’s *t* test or Kruskal–Wallis test were used to assess significance in phosphoproteomics data. Where applicable, *p*-values were adjusted for multiple testing using Benjamini–Hochberg method.

## Results

### Identification of Network Circuitry Associated With Synergism to PI3Ki Plus MEKi Cotreatment

**Fig. 1:**
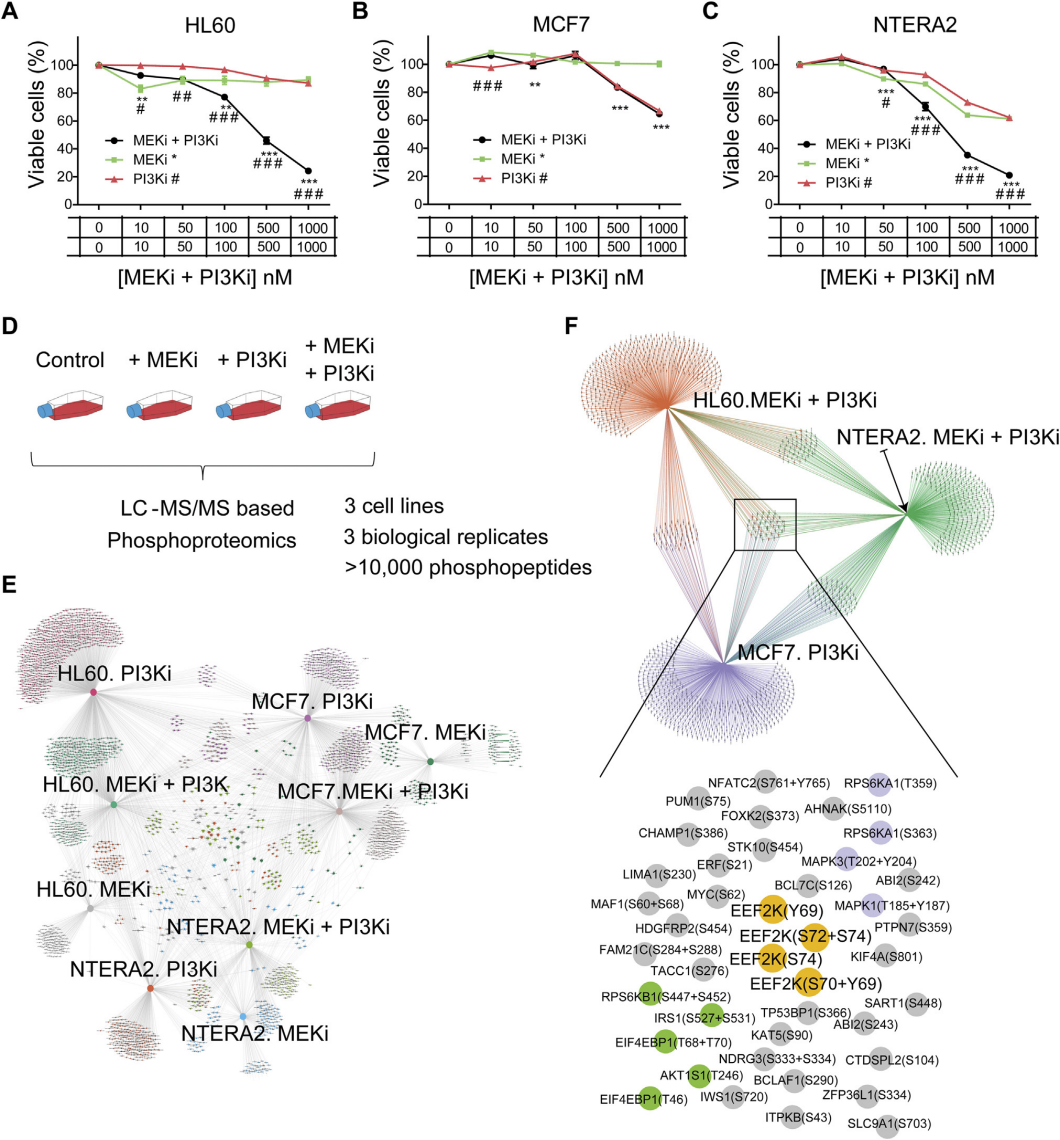
**Phosphoproteomics identifies downstream effectors of PI3Ki plus MEKi across three different cancer cell lines**. *A*–*C*, the named cell lines were treated with trametinib (MEKi) and/or pictisilib (PI3Ki) individually or in combination for 3 days at the indicated concentrations. Cell viability was measured using Guava ViaCount assays. Values indicate mean ± SEM (n = 3 independent experiments). Statistical significance was calculated by two-way ANOVA, ∗*p* < 0.05, ∗∗*p* < 0.01, ∗∗∗*p* < 0.001 (relative to MEKi), ^#^*p* < 0.05, ^##^*p* < 0.01, ^###^*p* < 0.001 (relative to PI3Ki). *D*, experimental design of the phosphoproteomic study. *E*, A 2-mode network constructed considering only phosphopeptides decreased with log_2_-fold change < -1 and *p*-value < 0.05. *F*, as in (E) but only selecting those conditions where cell viability was drastically reduced by treatment. Zoom shows phosphopeptides that decreased with statistical significance for all the indicated conditions.

To study how different cancer cell models respond to the combination of PI3K (GDC-0941, also known as pictilisib) and MEK inhibitors (trametinib), we first determined the synergism profiles of three cancer cell lines to cotreatment; namely, MCF7, NTERA2, and HL60, which are models derived from breast cancer, testicular carcinoma, and AML, respectively. We used inhibitor concentration ranges from 10 to 1000 nM, based on the IC50 *in vitro* of the compounds over the intended target. After 72 h treatment, individual compounds had a minor impact over HL60 cell viability, but a remarkable synergy (with cell viability lower than 50%) was observed when PI3Ki and MEKi were combined at 500 or 1000 nM each (Fig. 1*A*). In contrast, cotreatment in MCF7 cells was not synergistic, and the sensitivity to the PI3K inhibitor was identical to the cotreatment across all the concentrations tested (Fig. 1*B*). As with HL60, the cotreatment was synergistic in NTERA2 (Fig. 1*C*). Analysis of synergy using the coefficient of drug interaction (CDI) metric (22), confirmed that the cotreatment was synergistic in both NTERA2 and HL60 (CDI values below 1) from 100 nM to 1000 nM drug concentrations (supplemental Fig. S1*A*).

To investigate the core PI3K-MAPK network circuitry in these models, we perturbed PI3K-mTOR and MAPK signaling by treating cells with MEKi and PI3Ki individually or in combination. After 1 h treatment, the activity markers p-ERK1/2 for MAPK pathway and p-AKT, p-PRAS40, p-p70S6K, and p-4EBP1 for PI3K-mTOR pathway decreased as expected in all cell models. Of interest, contrary to the canonical understanding of PI3K and MAPK signaling pathways, p-ERK1/2 signal decreased in HL60 and MCF7 cells treated with the PI3Ki at 500 and 1000 nM (supplemental Fig. S2, *A* and *B*), indicating that MAPK pathway is downstream of PI3K in HL60 and MCF7 cells. Unexpectedly, in NTERA2 cells, we observed an increase of p-AKT, p-PRAS40, and p-p70S6K levels after MEKi treatment, indicating that the PI3K pathway is inhibited by the MAPK pathway in this model (supplemental Fig. S2*C*). Thus, perturbation experiments revealed different patterns of PI3K-MEK signaling network circuitry across the profiled cancer cell lines.

### eEF2K Mediates Synergism to PI3Ki Plus MEKi Cotreatment

To understand the mechanisms that mediate synergy to PI3Ki + MEKi cotreatment, we performed a global phosphoproteomics analysis in cells treated for 1 h with MEKi and PI3Ki at 500 nM individually or in combination (Fig. 1*D*). We selected this concentration because it was synergistic in both HL60 and NTERA2. Phosphoproteomics experiments were performed in three biological replicates and two technical replicates and required 72 LC-MS/MS runs. In total, we identified 11,868 phosphorylation sites which could be quantified using label-free methods, as reported previously (7, 18, 21, 23) (Fig. 1*E*, supplemental Fig. S1, *B* and *C*). We found phosphopeptides regulated only by one kinase inhibitor treatment in 1 cell line and phosphopeptides regulated by multiple kinase inhibitors across multiple cell lines (Fig. 1*E*).

**Fig. 2:**
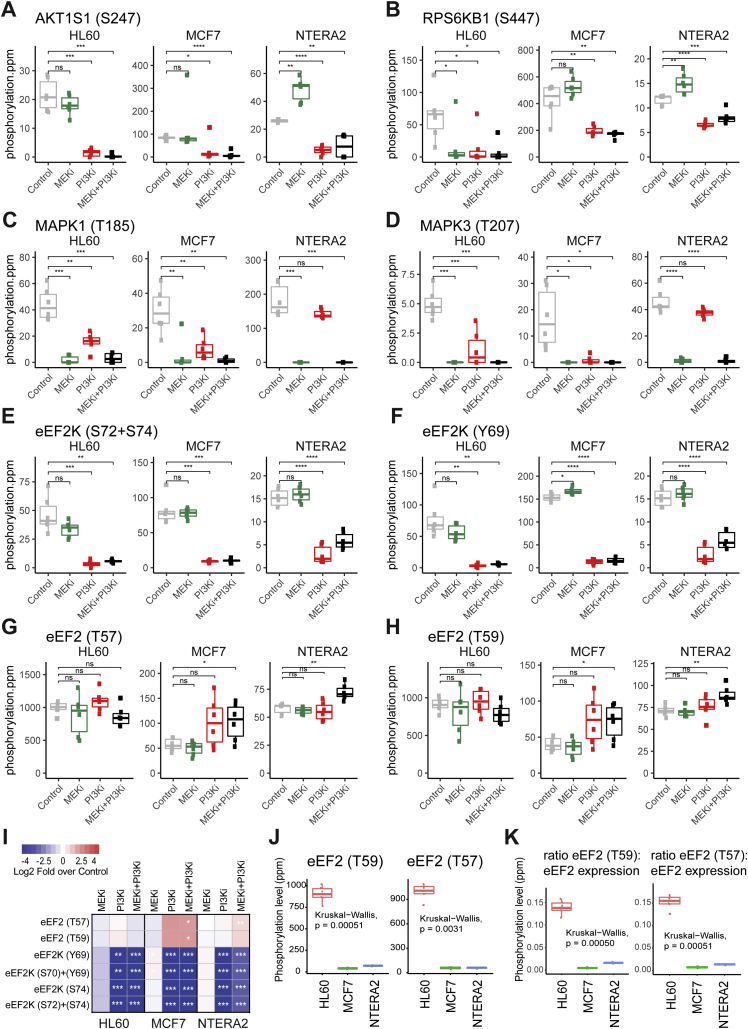
**Targeted analysis of PI3K/mTOR and MAPK pathway activity markers confirms regulation of eEF2K by PI3Ki plus MEKi cotreatment**. *A*–*H*, cancer cell lines were treated with kinase inhibitors for 1 h at 500 nM individually or in combination. Examples of phosphopeptide ion intensities (summed peak area) of phosphopeptides containing the named phosphorylation site across the named cell lines. Boxplots show median and interquartile ranges. *p*-values were calculated by *t* test (n = 3 independent experiments performed in technical replicates). Intensity values for each peptide were derived from the chromatographic peak areas of the extracted ion chromatograms and normalized to the total intensity in each sample. ppm denotes normalized intensity values multiplied by one million. *I*, heatmap of eEF2 and eEF2K phosphopeptides significantly modulated in at least one the conditions being compared to control. *p*-values were calculated by *t* test of log_2_ transformed data. ∗*p* < 0.05, ∗∗*p* < 0.01, ∗∗∗*p* < 0.001, ∗∗∗*p* < 0.0001. *J* and *K*, eEF2 phosphopeptide signals (J) and ratio of phosphorylation relative to eEF2 total protein expression levels (K) across all the named cell lines in basal conditions. *p*-values were calculated by Kruskal–Wallis test. eEF2, eukaryotic elongation factor 2; eEF2K, eukaryotic elongation factor 2 kinase.

Our strategy to identify phosphorylation sites that potentially mediate the synergy to the cotreatment was based on the data shown in Figure 1, *A*–*C*, which illustrates that treatment with MEKi+PI3Ki had a synergistic effect on the viability of HL60 and NTERA2 cells, while the PI3Ki on its own was able to reduce MCF7 cell viability. We then used our phosphoproteomics dataset (Fig. 1*D*) to construct a 2-mode network (2 types of nodes) containing phosphosites inhibited by the previously mentioned conditions (namely those that decrease cell viability in the three models tested, Fig. 1*F*). This analysis led to the identification of a group of phosphosites, included as nodes in the network, commonly affected by the three comparisons. These phosphopeptides included those in the canonical PI3K/AKT/mTOR pathway, such as sites in PRAS40 (AKT1S1), 4EBP1 (EIF4EBP1), IRS1 and p70S6K (RPS6KB1), and the MAPK pathway, such as ERK1 (MAPK3), ERK2 (MAPK1), and RSK1 (RPS6KA1). Interestingly, this group contained phosphorylations in Myc and p53 binding protein 1 (TP53BP1) that are also known to mediate cancer cell survival (24, 25), thus suggesting that this group of phosphorylation sites is enriched for regulators of cancer cell viability. To confirm these results, we carried out a targeted analysis of the identified phosphorylation sites across the assessed conditions, which confirmed the quality of the quantitative data (Fig. 2, *A*–*D*). Together, this analysis uncovered proteins and phosphorylation sites that potentially mediate synergy to PI3Ki + MEKi cotreatment.

Our attention focused on eEF2K phosphosites (Fig. 1*F*) because the activity of this kinase, a negative regulator of translation, is restricted by MAPK and PI3K pathways (26, 27). Thus, based on the known regulation of eEF2K, we expected to find an increase in eEF2K activity (detected as an increase of phosphorylated eEF2, the only known eEF2K substrate aside from autophosphorylation sites) as a result of inhibiting PI3K or MAPK pathways. Phosphoproteomics data showed that for all cell lines, eEF2K single (S74, Y69) and double phosphorylated forms (S72 + S74, S70 + Y69) were decreased in the presence of the PI3Ki, consistent with eEF2K being an mTORC1 substrate (26) (Fig. 2, *E* and *F*) and with previous findings showing that eEF2K phosphorylation sites decreased in cells treated with mTOR inhibitors, such as AZD8055, KU-0063794, and Torin 1 ((23) also see *chemphopro.org*). For two of the cell lines, NTERA2 and MCF7, the reduction of the inhibitory phosphorylation of eEF2K (at S74, Y69, S72 + S74, S70 + Y69) after drug treatment was associated with an increased phosphorylation of its substrate eukaryotic elongation factor 2 (eEF2) at T57 and T59 (Fig. 2, *G*–*I*). In HL60 cells, phosphorylation of eEF2 at T57 and T59 did not increase after treatment with PI3Ki, probably because in this cell line, eEF2 was highly phosphorylated at the basal level (Fig. 2*J*). This observation suggests a high basal eEF2K activity in HL60 cells, which is independent of eEF2 total expression levels (Fig. 2*K*). Therefore, PI3K and MEK blockade had no effect in the dephosphorylation (activation) of eEF2K in HL60 because this kinase was already highly active in these cells. Overall, our analysis identified cell type–specific regulation of eEF2K phosphorylation downstream of PI3K and MEK pathways.

**Fig. 4:**
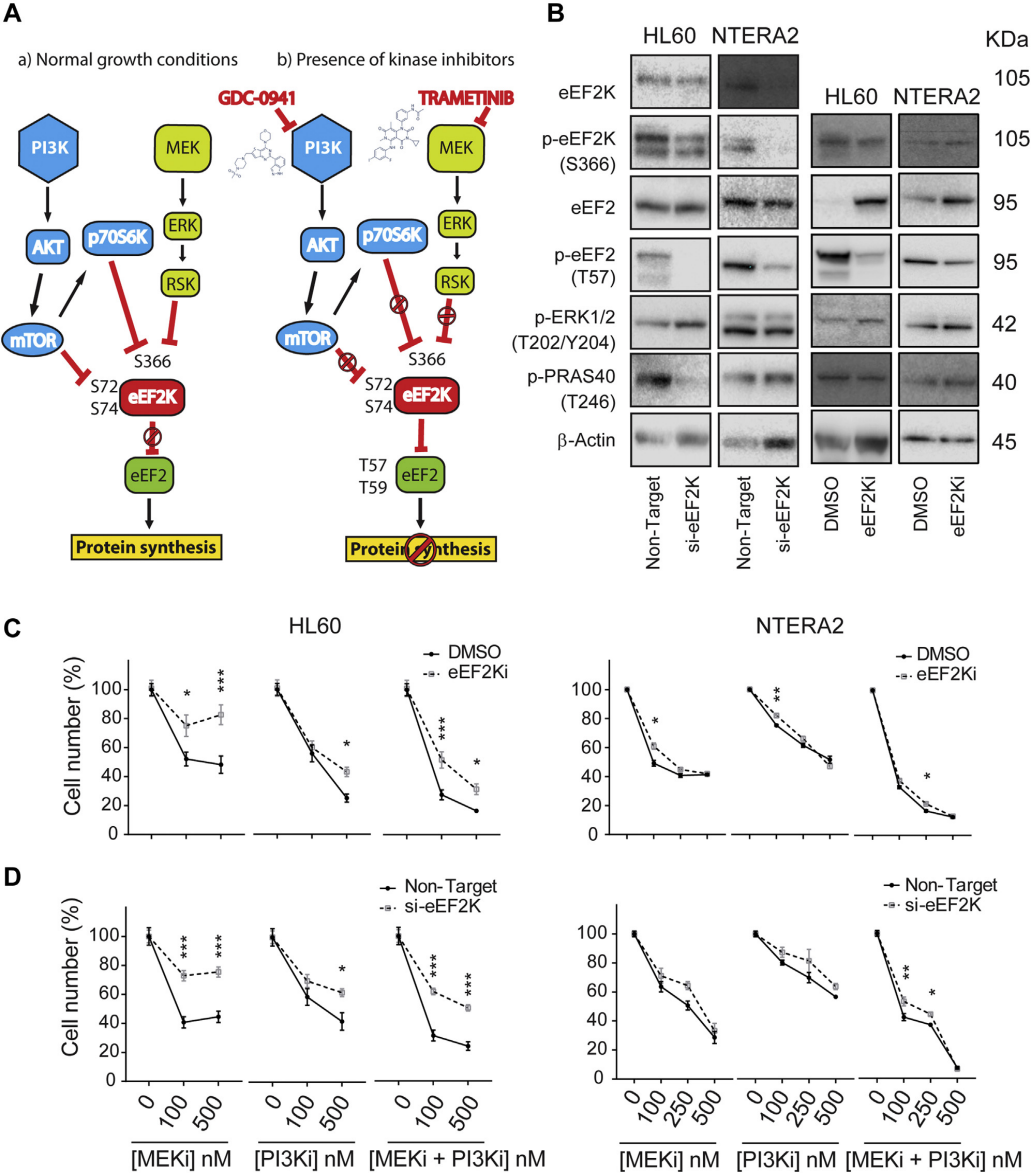
**eEF2K silencing by pharmacological or genetic means reverses the antiproliferative effects of PI3Ki plus MEKi cotreatment in cell models that responded synergistically to cotreatment**. *A*, scheme applied for NTERA2 cells. The signaling pathways PI3K/mTOR and MAPK negatively regulate eEF2K, thereby allowing for eEF2 activity to promote protein translation. With the individual addition of MEKi or PI3Ki into the cell culture, eEF2K still remains inactive, facilitating translation elongation. Only the combined therapy (B) leads to a full eEF2K activation and further eEF2 phosphorylation, slowing down the elongation stage of protein synthesis. *B*, phosphorylation of pathway activity markers in cells pretreated with an eEF2Ki for 24 h or transfected with nontarget siRNA or siRNA against *eEF2K* for 3 days. *C* and *D*, reduction in cell numbers (measured using a Guava assay) in cells in which eEF2K was silenced as in (*A*) and (*B*), followed by treatment with PI3Ki or MEKi individually or in combination for further 3 days at the concentrations shown. Values are mean ± SEM (n = 3 independent experiments). Statistical significance was calculated by two-way ANOVA, ∗*p* < 0.05, ∗∗*p* < 0.01, ∗∗∗*p* < 0.001. DMSO, dimethyl sulfoxide; eEF2, eukaryotic elongation factor 2; eEF2K, eukaryotic elongation factor 2 kinase; MAPK, mitogen-activated protein kinase; mTOR, mammalian target of rapamycin.

We hypothesized that eEF2K could be mediating synergism to PI3Ki + MEKi cotreatment in NTERA2 cells. We postulate that with individual inhibition of MEK or PI3K, eEF2K still remains inactive, facilitating translation elongation, whereas inhibition of both PI3K and MEK is required for full eEF2K activation and further eEF2 phosphorylation, needed for slowing down translation elongation. This model applies to NTERA2 cells (Fig. 4*A*), where the PI3K/MAPK network is wired in a way in which cotreatment with PI3Ki and MEKi is needed to inhibit both pathways, and in MCF7 where the inhibition of PI3K is sufficient to inhibit both MAPK and PI3K pathways. As noted above, eEF2K has high basal activity in HL60 cells, which cannot be increased by further dephosphorylation as it already is fully dephosphorylated.

**Fig. 3:**
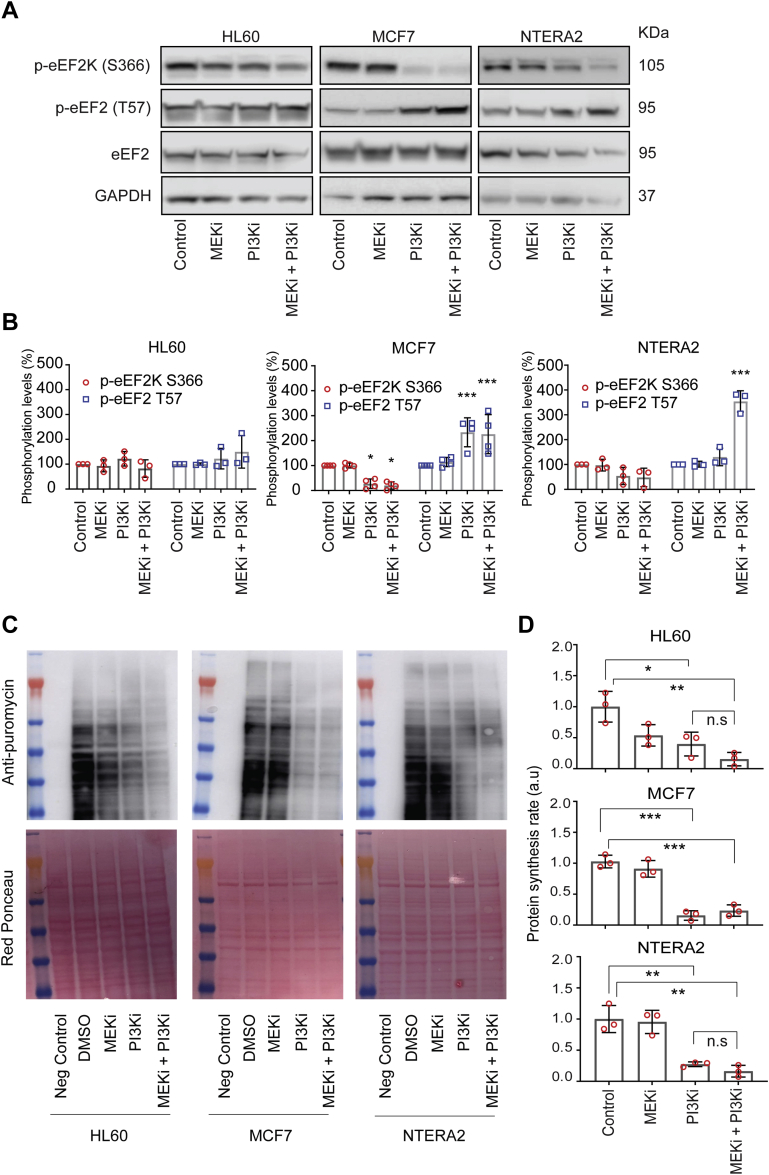
**PI3Ki and MEKi cooperate in the regulation of eEF2 phosphorylation and in the inhibition of protein synthesis**. *A*, eEF2 and eEF2K phosphorylation abundance across cell lines after treatment with 500 nM of PI3K and MEK inhibitors individually or in combination for 1 h. *B*, quantification of Western blot signals from eEF2 and eEF2K phosphorylation sites using densitometry data. Values are mean ± SEM (n = 3 independent experiments, n = 4 for MCF7). Statistical significance was calculated by two-way ANOVA, ∗*p* < 0.05, ∗∗∗*p* < 0.001. *C*, antipuromycin was probed to detect puromycin incorporation into *de novo* synthesized proteins as function of the indicated treatments (inhibitors were used at 500 nM concentration in the media). *D*, quantification of puromycin signal using densitometry data. Values are mean ± SEM (n = 3). Statistical significance was calculated by *t* test, ∗*p* < 0.05, ∗∗*p* < 0.01, ∗∗∗*p* < 0.001 (relative to control). DMSO, dimethyl sulfoxide; eEF2, eukaryotic elongation factor 2; eEF2K, eukaryotic elongation factor 2 kinase; MEK, MAPK/ERK kinase.

In order to confirm the validity of our model of synergy, which we derived based on phosphoproteomics observations, we checked (by immunoblotting) whether other phosphorylation sites on eEF2K and eEF2 are also regulated by PI3Ki and MEKi by themselves or in combination (Fig. 3, *A* and *B*). In NTERA2 cells, the drug combination was required to decrease eEF2K phosphorylation at S366 (a site that was not detected by LC-MS/MS) (Fig. 3, *A* and *B*, right panels). This site has been reported to be inhibitory and to be phosphorylated downstream of both mTORC1 and MAPK pathways (28). Decrease of eEF2K S366 phosphorylation led to a concomitant increase in phosphorylation of its substrate eEF2 (T57) by about 4-fold (Fig. 3*B*, right panel). Single treatment with PI3Ki in MCF7 cells was enough to reduce eEF2K phosphorylation by about 4-fold and increase eEF2 phosphorylation 2-fold (Fig. 3, *A* and *B*, middle panels), supporting again the impact of PI3Ki over both PI3K and MAPK pathways previously observed in this model. Consistent with high basal activity of eEF2K in HL60 cells, treating these cells with PI3Ki or MEKi produced modest and not significant changes of eEF2K activity markers (Fig. 3, *A* and *B*, left panels). These data suggest that synergy between PI3Ki and MEKi needs high eEF2 inactivation by phosphorylation to inhibit protein synthesis.

To study this possibility, we investigated whether an enhanced eEF2K activity (as a result of PI3Ki + MEKi cotreatment in NTERA2 cells) causes a slowdown of protein synthesis rate. To this end, we treated cells with PI3Ki and MEKi for 5 h, individually or in combination at 500 nM each. Cells were then treated with puromycin, an aminoacyl-tRNA analog that covalently attaches to the C terminus of *de novo* synthesized peptides during protein translation.

In MCF7 cells, the PI3Ki reduced the translation rate while the addition of MEKi did not have any effect. In HL60 and NTERA2 cells, the PI3Ki also inhibited protein synthesis. The combination of PI3Ki and MEKi did not significantly increase the effect of PI3Ki but when compared to control, the effect of PI3Ki+MEKi presented a higher magnitude and a significance than the individual treatments. Therefore, our data indicate a trend in which the combination of MEKi and PI3Ki produced a higher reduction of protein synthesis rate than any of the inhibitors alone in HL60 and NTERA2, while the MEKi did not have any effect on the protein synthesis rate of MCF7 cells (Fig. 3, *C* and *D*).

Interestingly, in cells that are susceptible to synergy to cotreatment (HL60 and NTERA2), the effect on protein synthesis trended to be higher than the single treatment, suggesting that PI3Ki + MEKi treatment is synergistic when it caused a decreased in protein synthesis greater than the single treatment alone.

### Genetic or Pharmacological Inhibition of eEF2K Rescue the Antiproliferative Effects of PI3Ki and MEKi

To evaluate whether eEF2K activity is implicated in the synergistic effect of PI3Ki + MEKi cotreatment, we inhibited eEF2K activity with either siRNA or a small molecule inhibitor (named A484954) which, based on its known target selectivity, is specific for eEF2K at the concentrations tested (supplemental Fig. S3*A*). This compound inhibits eEF2 phosphorylation but has no inhibitory effect on cell growth (29). In HL60 cells, p-eEF2 levels decreased after exposure of cells to the eEF2Ki for 24 h but not 6 h; we therefore selected 24 h for treatments with this compound in viability assays. Of interest, we found an increased eEF2K activity in MCF7 after 24 h (supplemental Fig. S3*A*). This effect, previously observed in other models, has been ascribed to nutrient depletion, and it is in agreement with the role of eEF2K protecting cells against nutrient deprivation (30).

eEF2K basal activity in HL60 cells decreased when cells were pretreated for 2 h with the eEF2Ki, confirming that this compound inhibits eEF2K activity after a short treatment time (supplemental Fig. S3*B*). Furthermore, eEF2K activity was increased in NTERA2 cells cotreated for 1 h with MEKi + PI3Ki at 100 nM each, and those cells pretreated with the eEF2Ki or siRNA-transfected cannot show an enhanced eEF2K activity after cotreatment (supplemental Fig. S3, *B* and *C*).

To investigate the mechanisms by which eEF2K modulates sensitivity to PI3Ki + MEKi, we analyzed pathway activity markers as a function of eEF2Ki treatment. This compound did not affect p-eEF2K abundance (Fig. 4*B*), but as expected, eEF2 phosphorylation levels decreased across cells pretreated with the eEF2Ki. We used p-ERK1/2 (MAPK1/3) and p-PRAS40 (also known as AKT1 substrate 1) as markers of MEK/MAPK and PI3K pathway activities, respectively, which were unaffected by the presence of the eEF2Ki. Similarly, as Figure 4*B* shows, a reduction of eEF2K expression by siRNA against *eEF2K*, was concomitant with a decreased in abundance of both p-eEF2K (S366) and the substrate p-eEF2 (T57). These data show successful target inhibition of eEF2K by siRNA and the small molecule chemical probe A484954.

Consistent with the notion that eEF2K mediates synergy to PI3Ki-MEKi cotreatment, we found that inhibition of eEF2K with A484954 (Fig. 4*C*) or siRNA transfection (Fig. 4*D*) reversed the antiproliferative effects of both PI3Ki and MEKi. This was observed when using the proportion of viability cells (supplemental Fig. S4, *A* and *B*, left panels) or absolute cell numbers (Fig. 4, *C* and *D*, left panels) as the endpoint of the pharmacological experiments: the effect was greater at 100 nM, where pretreatment of HL60 cells with eEF2Ki or siRNA rescued the antiproliferative effects of cotreatment by 50 and 60%, respectively (Fig. 4, *C* and *D*, left panels). eEF2K blockade rescued the antiproliferative effects of MEKi + PI3Ki in HL60 to a greater extent than in NTERA2 cells, in agreement with a higher eEF2K activity in the HL60 cell line relative to other models (Fig. 2*K*). These results indicate that cells with blocked eEF2K activity have a diminished capacity to respond to MEKi + PI3Ki. In NTERA2 cells, rescue to cotreatment was observed at 100 and 250 nM (exclusively in those conditions where eEF2K activity is high) but not at 500 nM, due to a drastic decrease in cell viability by treatment with single compounds (supplemental Fig. S4, *A* and *B*, right panels). A small increase in cell numbers was observed in NTERA2 cells treated with MEKi + PI3Ki in combination at 100 and 250 nM (Fig. 4, *C* and *D*, right panels). The reduction of eEF2K activity resulted in a decrease of p-eEF2 levels (Figs. 2 and 3) with concomitant enhancement of cell proliferation in PI3Ki or MEKi-treated cells (Fig. 4, *C* and *D*), thus suggesting that eEF2K play an essential role in mediating synergy to PI3Ki-MEKi and in the survival of cells that respond to the cotreatment.

### eEF2K Activity Predicts Synergy in AML Models

**Fig. 5:**
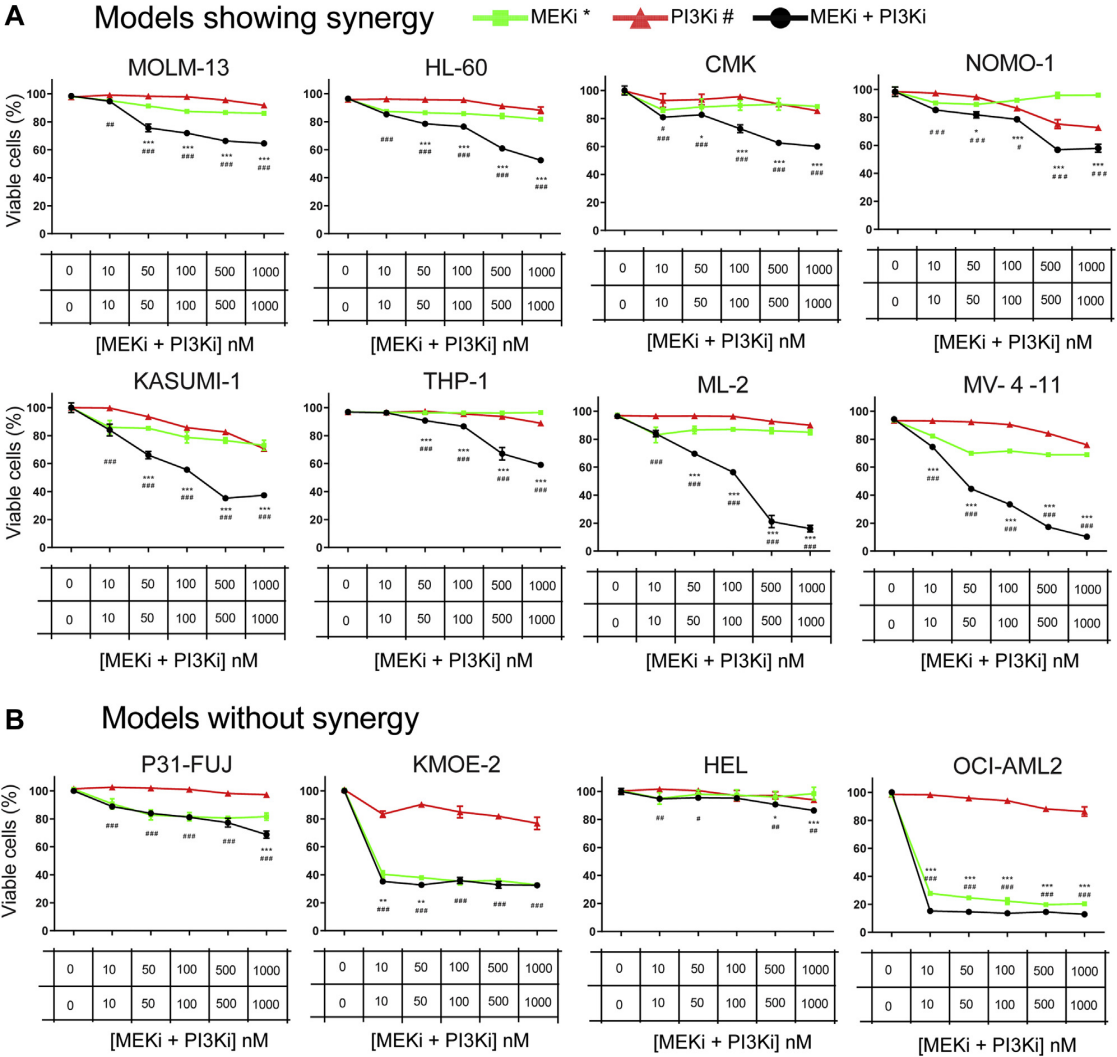
**AML cell lines show heterogeneous profiles of synergy to PI3Ki plus MEKi cotreatment**. *A* and *B*, cell viability was measured in 12 AML cell lines using a Guava ViaCount after treatment with the named kinase inhibitors individually or in combination for 3 days at the concentrations shown. Values indicate mean ± SEM (n = 3 technical replicates). Statistical significance was calculated by two-way ANOVA, ∗*p* < 0.05, ∗∗*p* < 0.01, ∗∗∗*p* < 0.001 (relative to MEKi), ^#^*p* < 0.05, ^##^*p* < 0.01, ^###^*p* < 0.001 (relative to PI3Ki). AML, acute myeloid leukemia.

Our results indicate that eEF2K activity mediates synergy to cotreatment in two out of three cell lines tested. We next sought to investigate the relationship between eEF2K activity and synergy to PI3Ki and MEKi treatment in a larger panel of cancer models. Since basal eEF2K activity in the leukemic cell line HL60 was higher than in the other cell lines (Fig. 2*J*), we decided to use 12 AML cell lines shown in Figure 5 as our model system. We first determined the synergy of PI3Ki and MEKi in individual AML cell lines treated for 3 days at drug concentrations ranging from 10 to 1000 nM. Based on drug response curves and CDI values, we found high synergy to cotreatment in eight cell lines: namely MOLM-13, HL60, KASUMI-1, THP-1, ML-2, MV4-11, CMK, and NOMO-1, (Figs. 5*A* and S5*A*); whereas, for HEL, OCI-AML2, P31/FUJ, and KMOE-2, the PI3Ki + MEKi treatment was not synergistic (Fig. 5*B*). CDI values for OCI-AML2 were below 0.9 at some concentrations (supplemental Fig. S5*B*), but the drastic decrease on cell viability when combined drugs was similar to the MEKi effects by itself (Fig. 5*B*). These data stablished two groups of AML cell lines based on whether PI3Ki + MEKi was synergistic in reducing cell viability (Fig. 5).

**Fig. 6:**
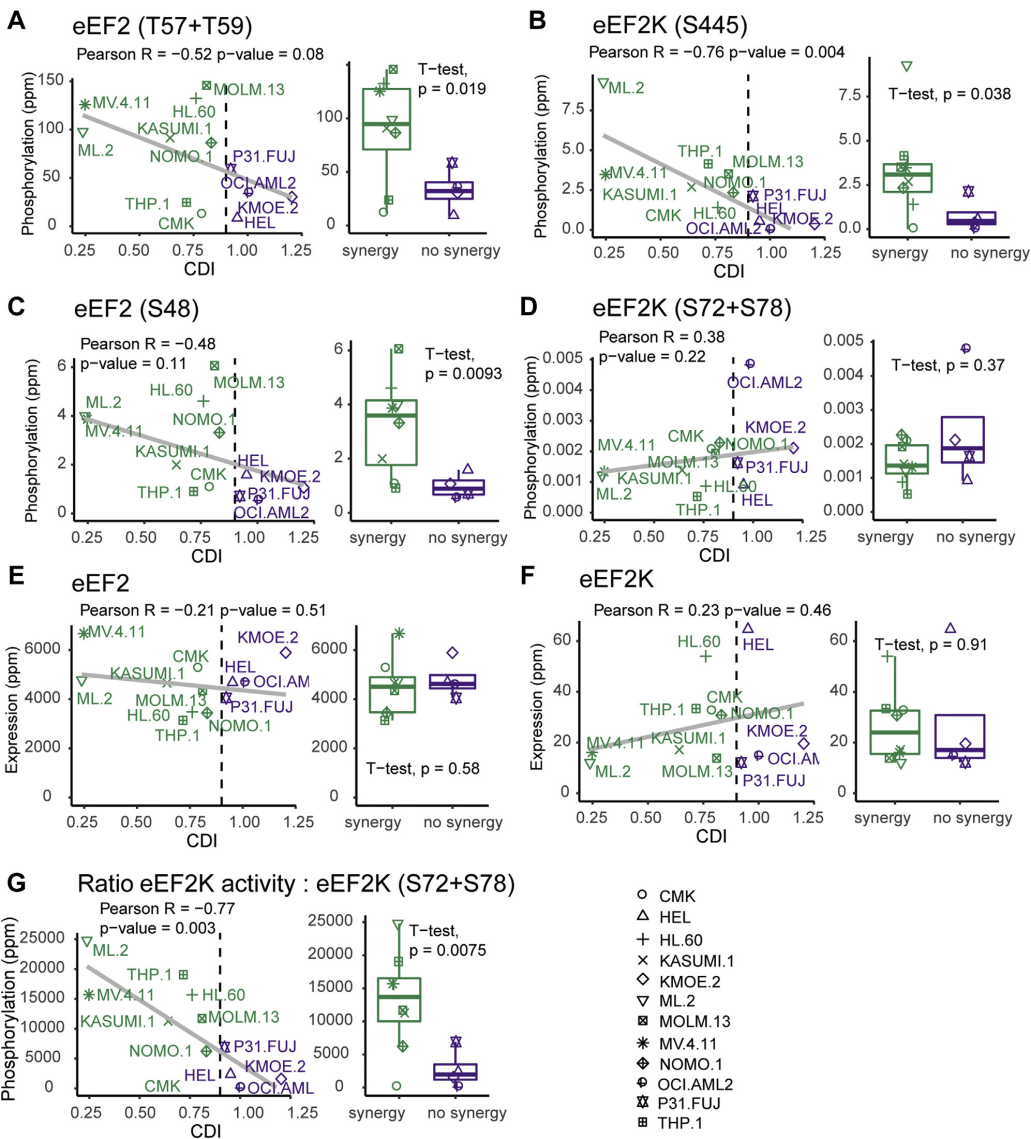
**Identification of an eEF2K activity signature significantly associated to the extent of PI3Ki plus MEKi synergy in a panel of 12 AML cell lines**. *A*–*F*, association of the named phosphorylation sites (*A*–*D*) and protein abundances (*E*–*F*) with averaged coefficient of drug interaction (CDI) values calculated from cells treated with MEKi and PI3Ki in combination at 500 and 1000 nM for 3 days. Data points in boxplots show median and interquartile ranges. *p*-values were calculated by Pearson and by a two-sided Wilcoxon test (n = 3 independent experiments performed in technical replicates). *G*, an eEF2K activity signature was determined as the ratio of phosphorylation extent of eEF2 at T57 + T59 plus eEF2K at S445 (direct eEF2K substrate and autophosphorylation site, respectively) relative to eEF2K phosphorylation at S72 + S78 (inhibitory sites). This activity signature was then correlated to CDI values as in (*A*–*F*). Statistical significance of the correlation was determined as in (*A*–*F*) intensity values for each peptide were derived from the chromatographic peak areas of the extracted ion chromatograms and normalized to the total intensity in each sample. ppm denotes normalized intensity values multiplied by one million. eEF2, eukaryotic elongation factor 2; eEF2K, eukaryotic elongation factor 2 kinase.

We next obtained basal phosphoproteomics and proteomics data for our cell line panel from (31) (PRIDE ID: PXD019591). Although these experiments were performed using data-dependent identification methods, we targeted the analysis to eEF2 and eEF2K peptides and phosphopeptides postacquisition by performing extracted ion chromatogram quantification of those peptides, as reported previously (7, 18, 21). Interestingly, we found that cells for which the PI3Ki + MEKi treatment was synergistic had an increased phosphorylation of eEF2 at T57/T59 and S48 and eEF2K at S445 (*p* < 0.05 by *t* test, Fig. 6, *A*–*C*). This latter site is an autophosphorylation site and a marker of kinase activation (32). In contrast, the abundance of eEF2K inhibitory sites at S72/S78 was not different between models (Fig. 6*D*).

To assess the relationship between eEF2K activation and synergy to PI3Ki + MEKi more systematically, we derived an eEF2K activity index—calculated by taking the ratio between the abundance of activating and inhibitory sites. This measure of kinase activity was higher in cells for which the cotreatment was synergistic and highly associated with CDI values (Pearson *p*-value = 0.003 and *t* test *p*-value = 0.0075, Fig. 6*G*). The association between phosphosite markers of eEF2K activity and CDI synergy values was not due to differences in eEF2 and eEF2K protein abundance (Fig. 6, *E* and *F*) and was further validated by immunoblotting (supplemental Fig. S6*A*).

We also investigated the relationship between the activation of PI3K and MAPK pathways and synergy to PI3K and MEK inhibitors. Expression levels of proteins involved in the MAPK pathway, such as ERK1 and MEK1 were similar between groups of cells showing differences in synergy to PI3Ki + MEKi (supplemental Fig. S7, *A* and *B*). However, components of other MAPK pathways, such as JNK1 and p38 delta isoform, and some translation initiation factors, including eIF5A2 and eIF6, were enriched in the “no synergy” group (supplemental Fig. S7, *C* and *F*). Consistent with an increased expression of mRNA-binding proteins involved in translation initiation, our results revealed an enhanced protein synthesis rate in the “no synergy” group detected as a higher puromycin incorporation (supplemental Fig. S6, *B* and *C*). Conversely, proteins involved in the PI3K pathway, including the PI3K isoform delta, p70S6K and NRAS, tended to be increased in the group of cells for which the treatment was synergistic, although the difference in expression was only statistically significant for NRAS (by Pearson and *t* test, supplemental Fig. S7, *G*–*I*). Differences in PI3K isoform delta, p70S6K, and ERK1/2 protein abundances across response groups were further confirmed by immunoblotting (supplemental Fig. S6*A*). Overall, these results indicate that the group of eight AML cell lines for which MEKi + PI3Ki cotreatment shows synergy presented higher phosphorylation on eEF2K activity markers (both eEF2 and eEF2K autocatalysis) as well as increased expression of PI3K pathway members and RAS. These sites and proteins may therefore represent a potential biomarker signature to predict MEKi + PI3Ki synergy in cellular models of AML.

**Fig. 7:**
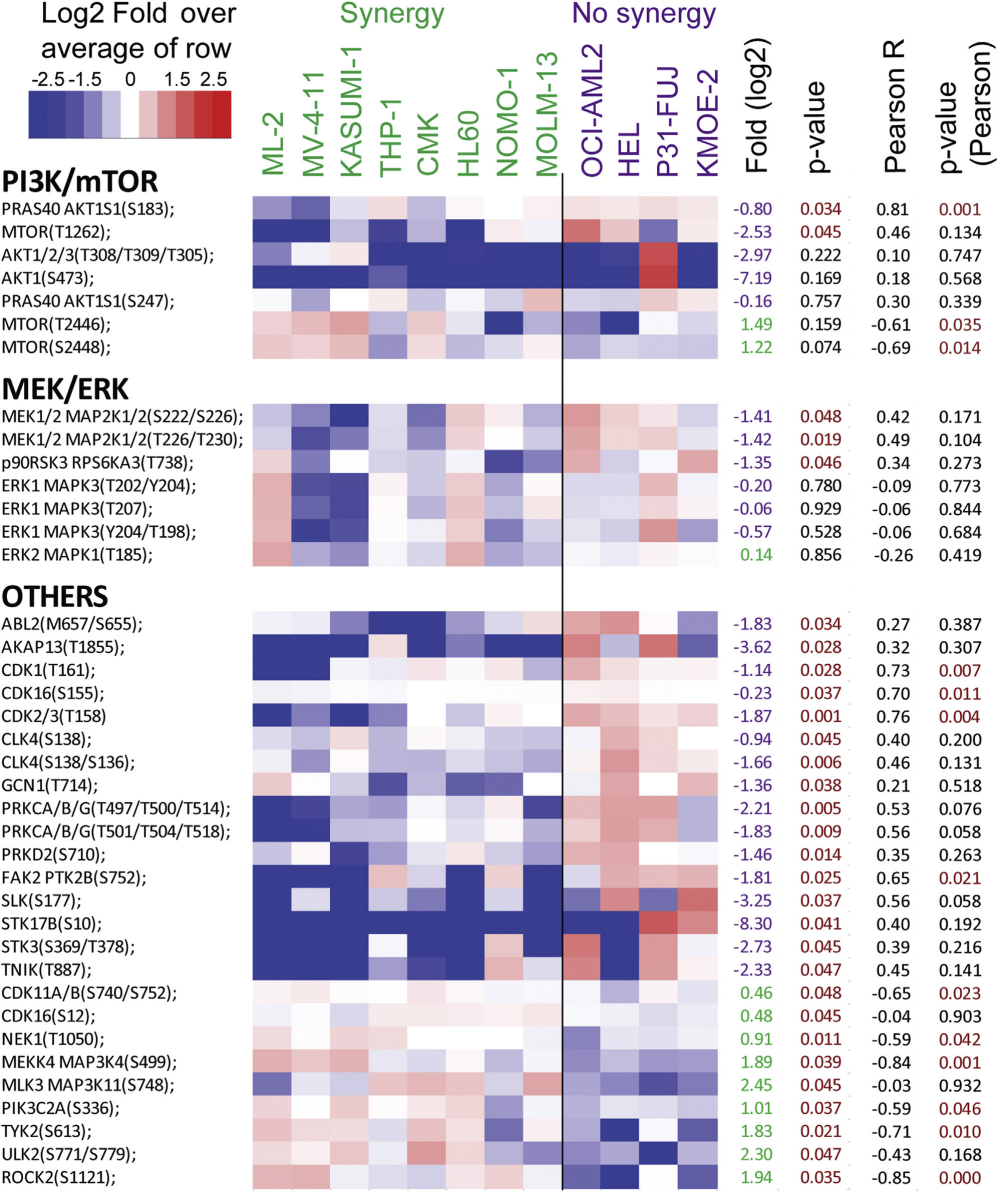
**Identification of phosphorylation sites associated to synergy of PI3Ki plus MEKi cotreatments in 12 AML cell lines**. Relative levels of phosphorylation sites in members of the named signaling pathways. Fold changes (in log_2_ scale) were calculated over the row mean and *p*-values were calculated by *t* test of log_2_ transformed data. AML, acute myeloid leukemia.

From the phosphoproteomics data, we also found some mTOR activity markers to be increased in the “no synergy” group, including PRAS40 (at S183) and mTOR phosphorylation at S1261 (Fig. 7), as site that promotes mTORC1-mediated substrate phosphorylation (33). This is consistent with the abundance of the eEF2K inhibitory site at S78, an mTORC1 substrate (34) which is slightly increased in the “no synergy” group (Fig. 6*D*). Additionally, we also found that the phosphorylation of mTOR at T2446 and S2448, which is catalyzed by S6K (35), was increased in the “synergy group” (Fig. 7). This could indicate the presence of negative feedback loops that restrain the activity of the pathway. Thus, several lines of evidence support the notion that mTOR activity is enhanced in the “no synergy” group, whereas MEK/ERK activity was similar across groups (Fig. 7). Interestingly, we found different signaling patterns of phosphorylation on several kinases including cyclin-dependent kinase, CDC-like kinase 4, Abelson murine leukemia viral oncogene homolog 2, TRAF2, and NCK-interacting protein kinase and PKC among others, which suggest an overall increased activity of several kinase-driven pathways in the “no synergy” group. Treatments with the multitargeted kinase inhibitor midostaurin (which inhibits PKC and several other kinases in this group) produced weakly synergistic effects in HEL cells and an antagonistic response in P31/Fuj (supplemental Fig. S8). These observations suggest that these kinases have the potential to interact, compensate, and enhance signaling downstream PI3K and MEK in a cell type–specific manner and illustrate the complex interactions between these kinases in the network. In summary, differences in expression patterns of proteins involved in PI3K, mTOR, and MAPK pathways, as well as kinase activity markers, ultimately explain why AML cell lines respond to the cotreatment in a distinct manner.

## Discussion

Several kinase inhibitors of the PI3K-mTOR and MAPK signaling pathways are under different stages of clinical development, while others, like alpelisib (BYL-719) and trametinib, are already approved by the FDA to treat specific subgroups of cancer patients (36). Clinical trials evaluating pictilisib (GDC-0941) have not advanced; however, other pan and isoform specific PI3K inhibitors, such as copanlisib (BAY80–6946), duvelisib (IPI-145), and idelalisib (CAL-101) have been approved by the FDA for the treatment of relapsed and refractory follicular lymphoma (37, 38, 39). However, unfortunately, despite several decades of research and huge cost, most of these compounds are, in most cases, ineffective when used as single treatments. This less than expected success of inhibitors targeting kinases in the PI3K and MAPK networks can in part be attributed to the existence of pathways that compensate for target inhibition (8). Consequently, several clinical trials have been designed to evaluate combination therapies that simultaneously target PI3K and MAPK pathway members in order to prevent the occurrence of therapeutic resistance (40). For example, a clinical trial is ongoing to determine the safety profile and tolerability of alpelisib and trametinib given in combination in patients with aggressive and refractory meningiomas (NCT03631953). This strategy repurposes drugs that are already approved to treat different types of cancer, and it is based on the idea that cotreating compensatory pathways reduces the likelihood of cancers finding escape routes that lead to therapeutic resistance. However, the mechanisms that mediate synergy to combination therapies with PI3K and MAPK inhibitors are not well understood, and this impede the selection of the most appropriate cohort of patients to be treated with these drug combinations.

In this study, we aimed to identify mechanisms that explain why PI3K and MEK inhibitors are synergistic in reducing cancer viability in some but not all cancer cell models (Fig. 1). Our initial phosphoproteomics screens identified eEF2K to be potentially implicated in mediating synergism, and thus, we were prompted to study its role in this process (Fig. 2). eEF2K, a known convergence point for PI3K and MAPK pathways, is an atypical calmodulin-dependent protein kinase, which phosphorylates and inhibits eEF2, the protein that mediates the movement of ribosomes along mRNAs from one codon to the next during the elongation stage of translation. Hence, eEF2K acts as a negative regulator of protein synthesis and thus cell growth (28).

Wang *et al.* found that the mTORC1 and MAPK pathways cooperate to restrict eEF2K activity: mTORC1 signaling promotes the phosphorylation of eEF2K at S78 and S396 *in vitro* (26), both the stress kinase p38 delta (MAPK13 gene name) and mTORC1 can phosphorylate eEF2K at S359 (41, 42). Phosphorylation of S78 is sensitive to mTORC1 inhibition *in vivo* (34); however, it is less clear what contribution mTOR signaling makes to S396 phosphorylation. Our phosphoproteomics analysis add to this body of knowledge as it indicates that mTORC1 modulates additional phosphorylation sites in eEF2K, such as Y69, S70, S72, and S74, which is consistent with our previous findings (23). It is known that ribosomal protein S6 kinase (p70S6K) and RSKs phosphorylate eEF2K at S366 in the linker region (28). All phosphorylation sites mentioned are inhibitory sites, which promote eEF2K inactivation, eEF2 de-phosphorylation, and an increase in protein synthesis. In addition to these well-characterized sites, eEF2K contains other phosphorylation sites of unknown function in its sequence (namely, eEF2K at S18, S27 and S31), and these are not yet associated to specific kinases (see *chemphopro.org*). Thus, eEF2K activity shows a complex mechanism of regulation by several upstream pathways which is not yet fully elucidated.

Cells possess mechanisms to inhibit eEF2K to allow protein synthesis to proceed. Thus, it was suggested that inhibition of eEF2K may be therapeutically beneficial as this would impair cancer cell survival (43, 44). Here, we found that synergy between PI3Ki and MEKi depends on eEF2 inactivation by phosphorylation, an event that leads to a slowdown in protein synthesis. Our data suggest that cells with high basal eEF2K activity (such as in HL60) show synergy to cotreatment because, in these cells, inhibition of PI3Ki and MEKi cannot lead to further eEF2K dephosphorylation (activation), which represses translation. In this model, protein synthesis proceeds independently of eEF2. Interestingly, NTERA2 cells (for which the cotreatment is synergistic) have an enhanced eEF2K activity as a function of cotreatment (Fig. 3). These data suggest that the cotreatment is synergistic in HL60 and NTERA2 cells because the decrease in protein synthesis by such cotreatment is greater than by single treatments. However, MCF7 cells did not show synergy because PI3Ki inhibits both PI3K and MEK pathways, thus causing increased p-eEF2 by itself.

Rescue experiments provided functional evidence of the role of eEF2K in mediating synergy to PI3Ki + MEKi cotreatment. Indeed, eEF2K genetic depletion or its pharmacological inhibition rescued the antiproliferative effects induced by PI3Ki + MEKi cotreatment in HL60 cells and, to a lesser extent, in NTERA2 (Fig. 4). The larger viability rescue observed in HL60 is consistent with these cells having higher eEF2K than other models (Fig. 2*K*). The synergistic effect observed in these two models is dependent on eEF2 phosphorylation, since its inhibition *via* siRNA or eEF2Ki rescued the reduction in cell numbers (and cell viability) caused by the cotreatment. High levels of eEF2 phosphorylation are due to an increase in eEF2K activity elicited by the cotreatment (NTERA2) or because of a high basal eEF2K activity (HL60). Thus, our data suggest that high intrinsic activity of eEF2K and further eEF2 phosphorylation could serve as markers of synergy to PI3Ki + MEKi.

We then explored whether the PI3Ki + MEKi cotreatment was synergistic in a panel of AML cell lines (Fig. 5). Phosphoproteomic analysis of untreated AML cell lines identified several eEF2K activity markers (including p-eEF2 at T57/T59 and its autophosphorylation site S455) as a potential signature to predict responses to dual PI3Ki + MEKi treatment (Fig. 6). Hence, a potential approach for patient selection to therapies with PI3Ki + MEKi may involve measuring eEF2K basal activity by analyzing key phosphorylation markers on eEF2K and its downstream target eEF2.

Interestingly, we also found that cells for which PI3Ki + MEKi cotreatment was not synergistic increased the activity of mTOR and several other kinases including cyclin-dependent kinase, CDC-like kinase 4, Abelson murine leukemia viral oncogene homolog 2, TRAF2 and NCK-interacting protein kinase, and PKC among others. These kinases—which act in parallel or downstream of PI3K and MEK —possess the ability, at least in principle, to provide proliferative signals when PI3K and MAPK pathways are inhibited and thus explain the absence of response to cotreatment in these models (Fig. 7).

In summary, we found that the activity of eEF2K helps rationalizing the extent by which PI3Ki + MEKi cotreatment synergize in reducing cancer cell survival and proliferation. Thus, this marker could be of utility in the implementation of therapies that target PI3K/mTOR and MEK/MAPK pathway members in combination.

## Data Availability

The mass spectrometry phosphoproteomics data have been deposited to the ProteomeXchange Consortium *via* the PRIDE (45) partner repository with the dataset identifier PXD018873. Immunoblotting raw data have been deposited to Mendeley Data repository with the following https://doi.org/10.17632/xjsdxnpxkb.1.

## Supplemental data

This article contains supplemental data (31).

## Conflict of interests

P. R. C. is a co-founder of Kinomica Ltd. The other authors declare no competing interests.

## Supplemental Data

Hijazi et al supplementary material.docx

Dataset 1 Peptides identified_V3.xlsm
