# Supplementary material for: eEF2K Activity Determines Synergy to Cotreatment of Cancer Cells With PI3K and MEK Inhibitors
Source: Mol Cell Proteomics. 2022 May 2;21(6):100240. doi: 10.1016/j.mcpro.2022.100240 (PMC9184568; doi:10.1016/j.mcpro.2022.100240)
Supplement: Hijazi et al supplementary material.docx [file mmc1.docx]

**Supplementary Materials**

eEF2K activity determines synergy to co-treatment of cancer cells with PI3K and MEK inhibitors

Maruan Hijazi, Pedro Casado, Nosheen Akhtar, Saul Alvarez-Teijeiro, Vinothini Rajeeve, Pedro R. Cutillas

Table of Contents

1. Supplementary Datasets

- Supplementary Dataset 1. Phosphopeptides identified

1. Supplementary Figures

Figure S1. Impact of PI3Ki plus MEKi co-treatment in cell viability and in the phosphoproteomes of cancer cell lines

Figure S2. Analysis of MEK/ERK and PI3K/AKT/mTOR activity markers as a function of PI3Ki plus MEKi co-treatments reveals differences in network circuitry across cancer cells

Figure S3. Impact of eEF2K inhibition and silencing in the expression of eEF2K, ERK and AKT activity markers as a function of PI3Ki and MEKi treatments

Figure S4. Viability of cells pre-treated with a highly selective eEF2Ki or transfected with siRNA against *eEF2K* in cell models that responded synergistically to co-treatment

Figure S5. Extent of PI3Ki + MEKi synergism in a panel of 12 acute myeloid leukemia (AML) cell lines

Figure S6. Expression of selected signalling markers and protein synthesis in 12 AML cell lines in basal conditions

Figure S7. Protein abundance levels linked to coefficient of drug interaction (CDI) in a panel of 12 AML cell lines in basal conditions

Figure S8. Extent of PI3Ki, MEKi and PKCi synergism in P31-FUJ and HEL cells.

3. Supplementary Table (Resources Table)

**
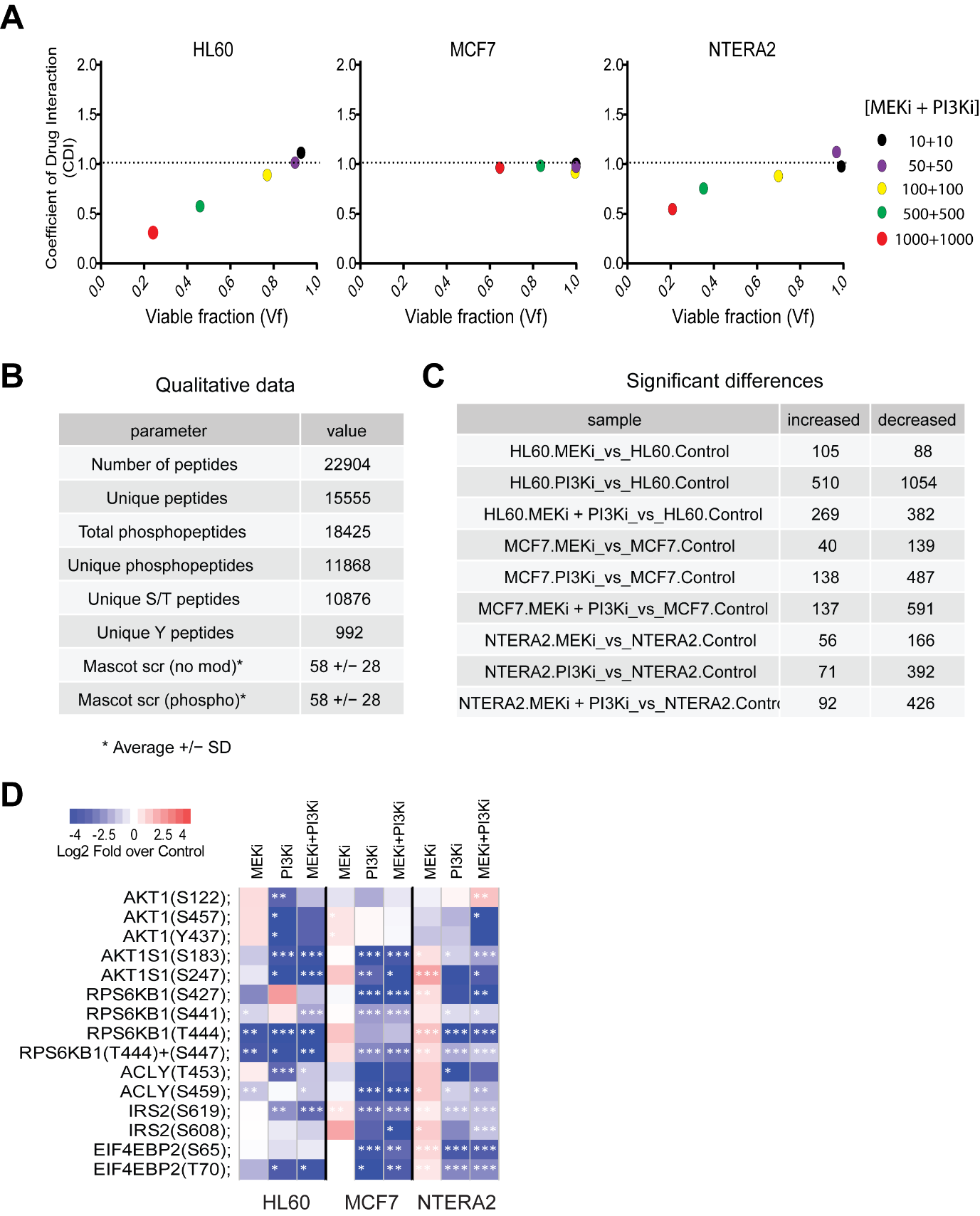
**

**Figure S1. Impact of PI3Ki plus MEKi co-treatment in cell viability and in the phosphoproteomes of cancer cell lines**

**(A)** Coefficient of drug interaction (CDI) values as a function of viability across cancer lines treated with the kinase inhibitors in combination at different concentrations. CDI was calculated as follows: CDI = AB/ (AxB), where AB is the ratio of the combination group to control group and A or B is the ratio of the single drug group to the control group. Values <1, =1 or >1 indicated that the drugs are synergistic, additive or antagonistic, respectively.

**(B)** Qualitative data of phosphoproteomics analysis.

**(C)** Significant differences across samples in phosphoproteomics analysis (at Benjamini-Hochberg adjusted p < 0.05 and fold change +/- 1).

**(D)** Heatmap of PI3K/AKT/mTOR activity markers significantly modulated in at least one the conditions being compared to control. P-values were calculated by t-test of log_2_ transformed data. *p<0.05, **p<0.01, ***p<0.001.

**
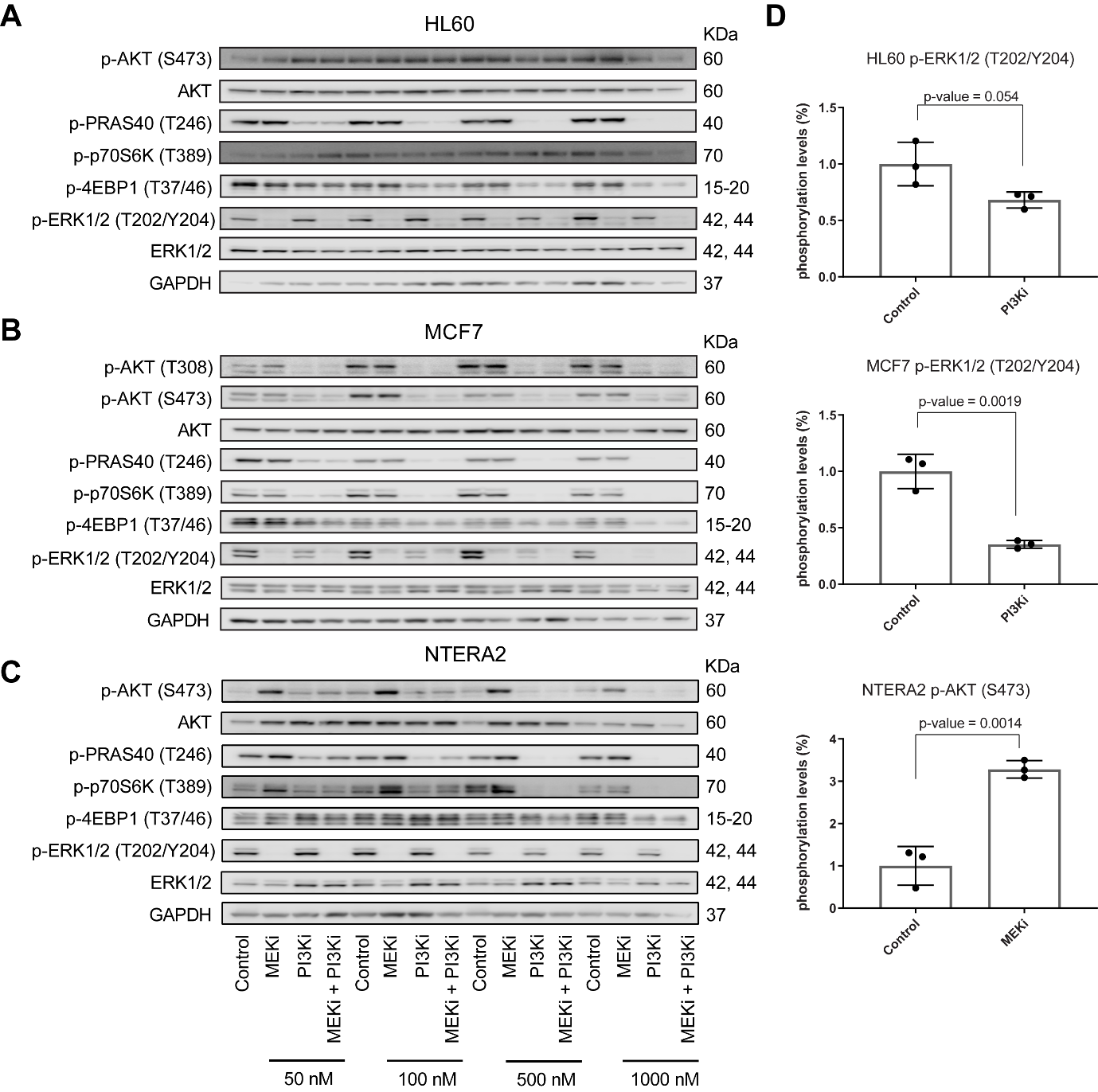
Figure S2. Analysis of MEK/ERK and PI3K/AKT/mTOR activity markers as a function of PI3Ki plus MEKi co-treatments reveals differences in network circuitry across cancer cells**

**(A, B, C)** Antibodies against the named markers of the PI3K/AKT/mTOR and MEK/ERK pathways were probed after treatments for 1 h with the named kinase inhibitors individually or in combination at the concentrations shown. The results revealed three different network circuitries across the three cell lines analysed.

(D) Quantification of Western blot signals from ERK1/2 and AKT phosphorylation sites using densitometry data. Values are mean ± SEM (n=3). Statistical significance was calculated by t-test.


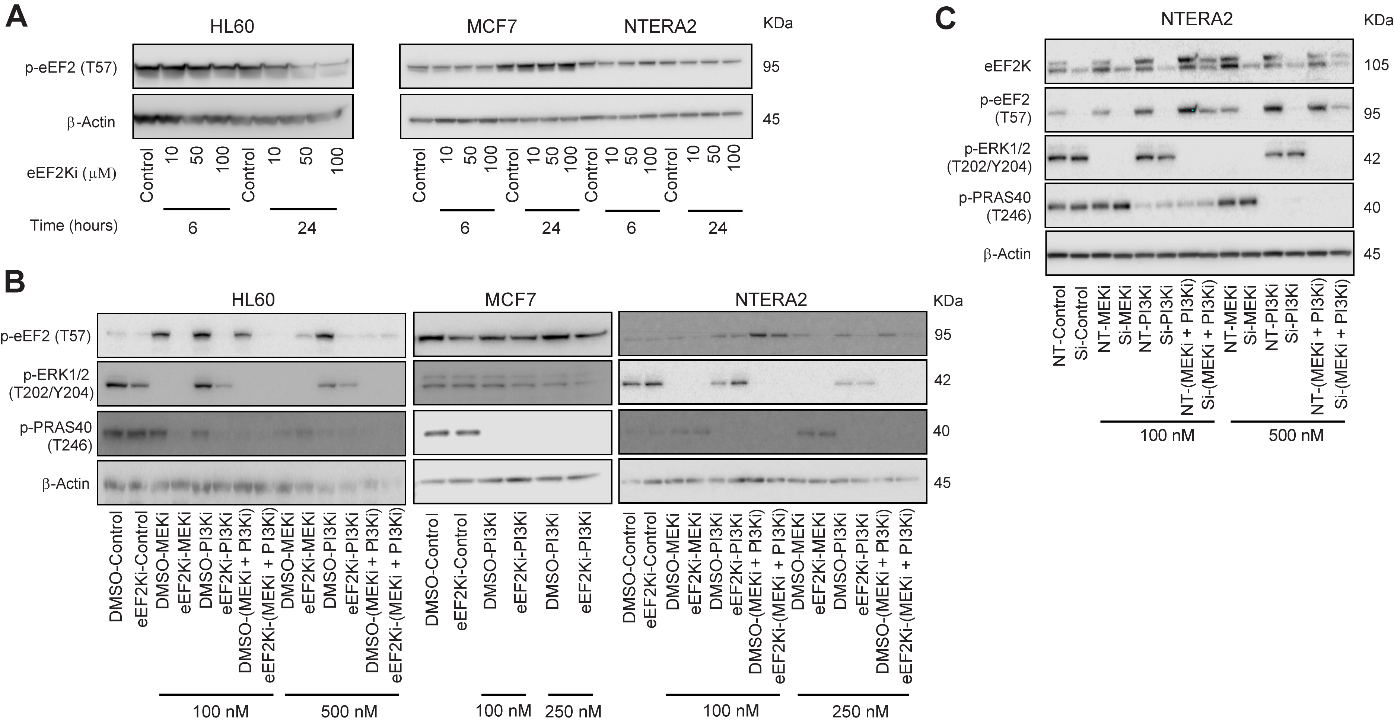


Figure S3. Impact of eEF2K inhibition and silencing in the expression of eEF2K, ERK and AKT activity markers as a function of PI3Ki and MEKi treatments

(A) Western blot analysis of eEF2 phosphorylation levels as a function of treatment with the eEF2Ki at the concentrations shown for 6 and 24 h. Inhibitor concentration used in HL60 cells was 50 µM, whereas in NTERA2 and MCF7 cells was 10 µM.

(B) Western blot analysis of eEF2K, ERK and AKT activity markers in cells pre-treated for 2 h with the eEF2Ki followed by treatment with the PI3Ki or MEKi individually or in combination for 1 h more at the indicated concentrations.

(C) Expression of eEF2K, ERK and AKT activity markers in NTERA2 cells transfected with siRNA against *eEF2K* for 3 days and then treated for 1 h with PI3Ki or MEKi individually or in combination at the concentrations shown.


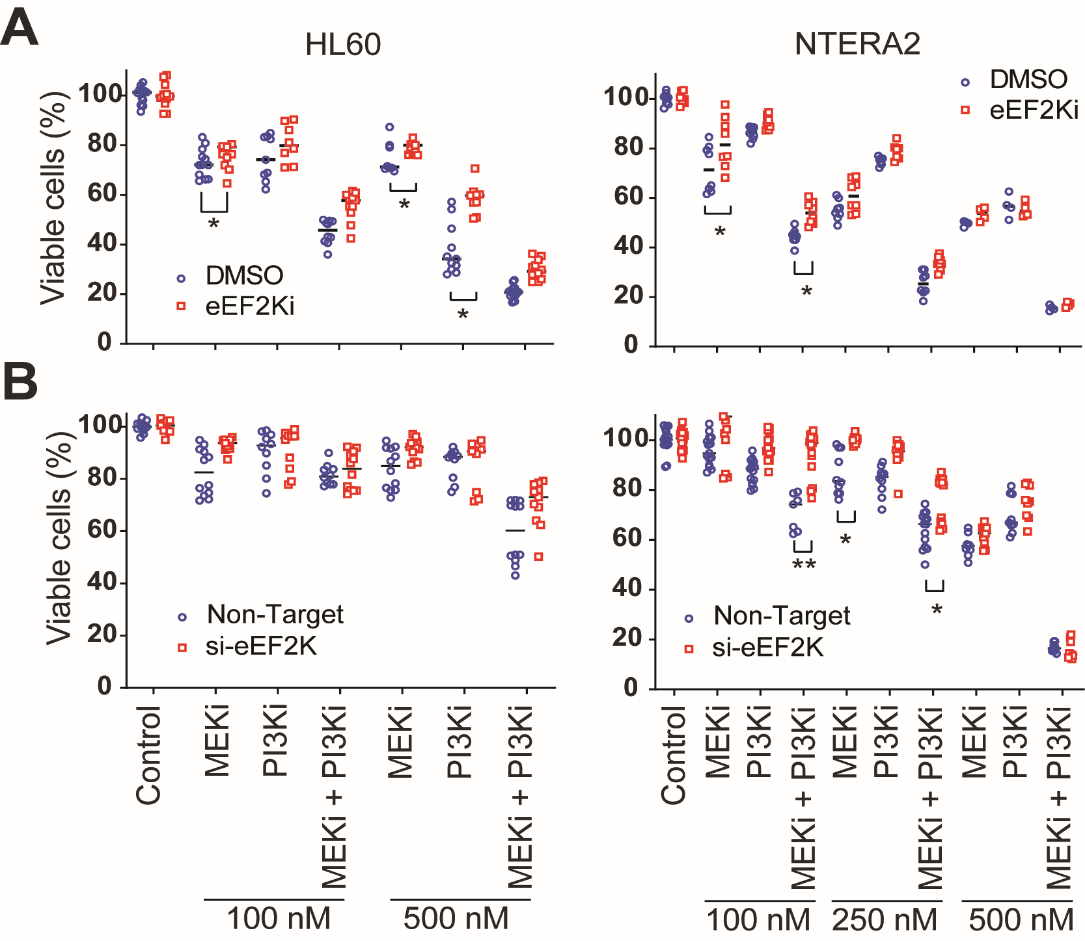


Figure S4. Viability of cells pre-treated with a highly selective eEF2Ki or transfected with siRNA against *eEF2K* in cell models that responded synergistically to co-treatment.

(A) Cell viability was measured using a Guava assay in cells pre-treated with an eEF2Ki for 24 h and further treated with PI3Ki or MEKi individually or in combination for 3 days.

(B) Cell viability in cells transfected with Non-Target siRNA or siRNA against *eEF2K* and treated with the named kinase inhibitors individually or in combination for 3 days at the concentrations shown. siRNAs were transfected 3 days before kinase inhibitor treatment. Values indicate mean ± SEM (n=3 independent experiments). Statistical significance was calculated by two-way ANOVA, *p<0.05, **p<0.01.


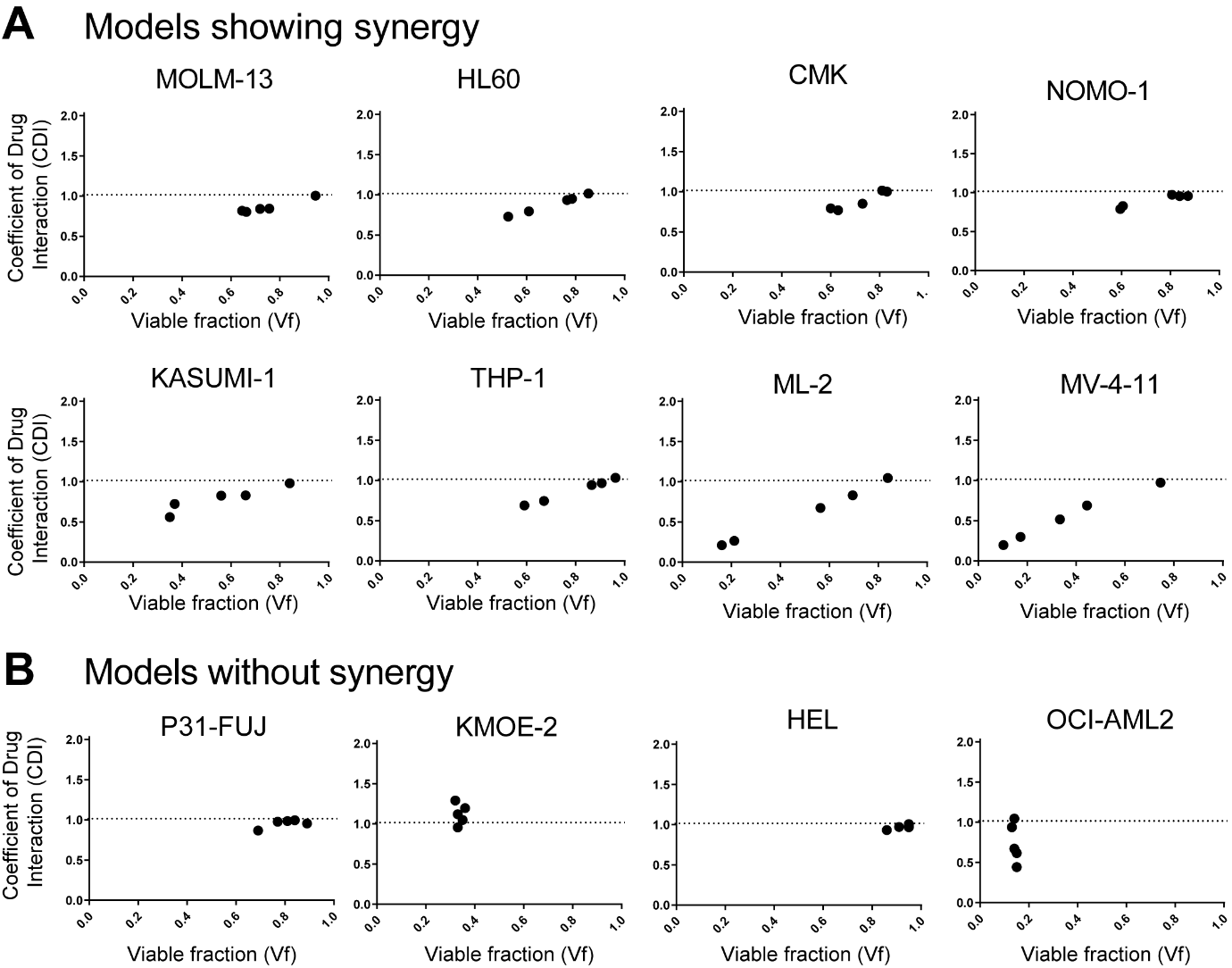


Figure S5. Extent of PI3Ki + MEKi synergism in a panel of 12 acute myeloid leukemia (AML) cell lines

CDI values as a function of viability in AML cell lines of which PI3Ki + MEKi co-treatment is synergistic **(A)** or no synergistic **(B)** at different concentrations. CDI was calculated as in Figure S1.


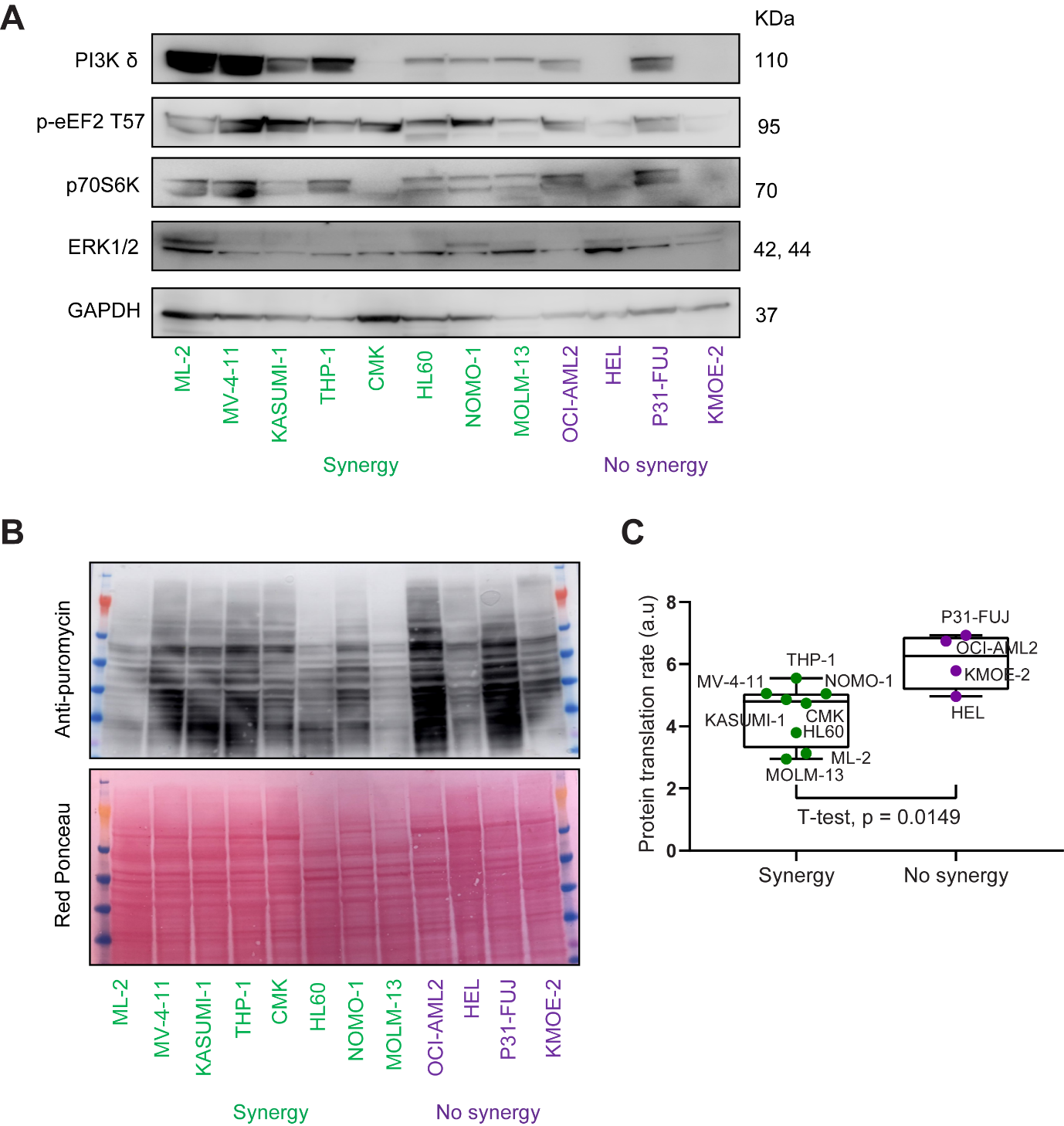


**Figure S6. Expression of selected signalling markers and protein synthesis in 12 AML cell lines in basal conditions**

(A) Western blots analysis of selected phosphoproteins and proteins in a panel of 12 AML untreated cell lines.

(B) Anti-puromycin was probed to detect puromycin incorporation into *de novo* synthesized proteins. Signal intensity correlates with protein synthesis during translation.

(C) Quantification of puromycin signal using densitometry data. Boxplots show median and interquartile ranges. P-value was calculated by t-test (n=6 performed in two independent experiments).

**
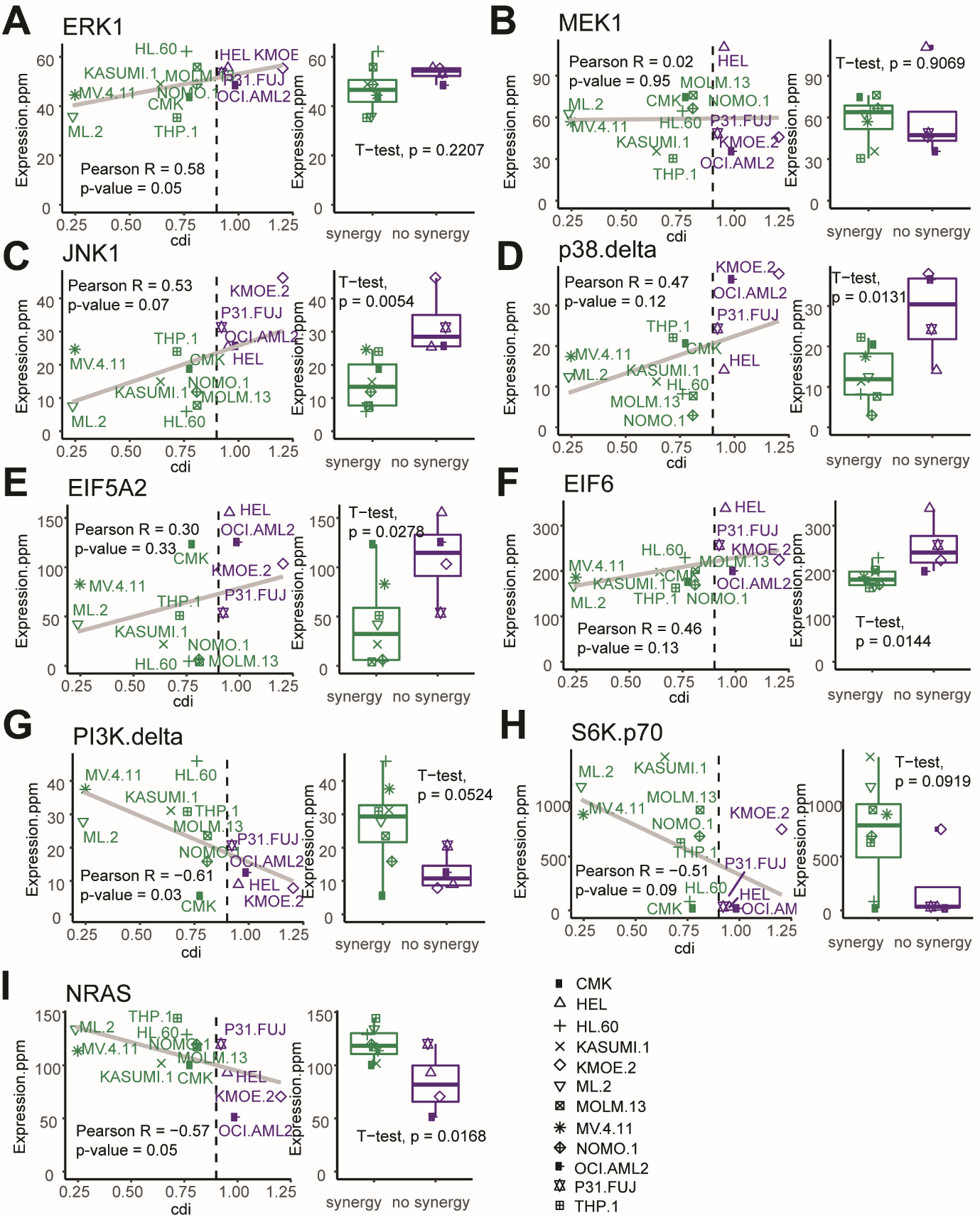
**

**Figure S7. Protein abundance levels linked to coefficient of drug interaction (CDI) in a panel of 12 AML cell lines in basal conditions.**

**(A-I)** Correlation values between protein abundances and averaged CDI values were calculated. CDI values were derived from cells that were treated with MEKi and PI3Ki in combination at 500 and 1000 nM for 3 days (R and P values were calculated by Pearson, n = 12). Correlations show different patterns of protein levels between “synergy” (denoted in green) and “non-synergy” (purple) models. Data points in boxplots show median and interquartile ranges. P-values were calculated using a two-sided Wilcoxon test (n=4 and 8 for “no synergy” and “synergy” models, respectively).


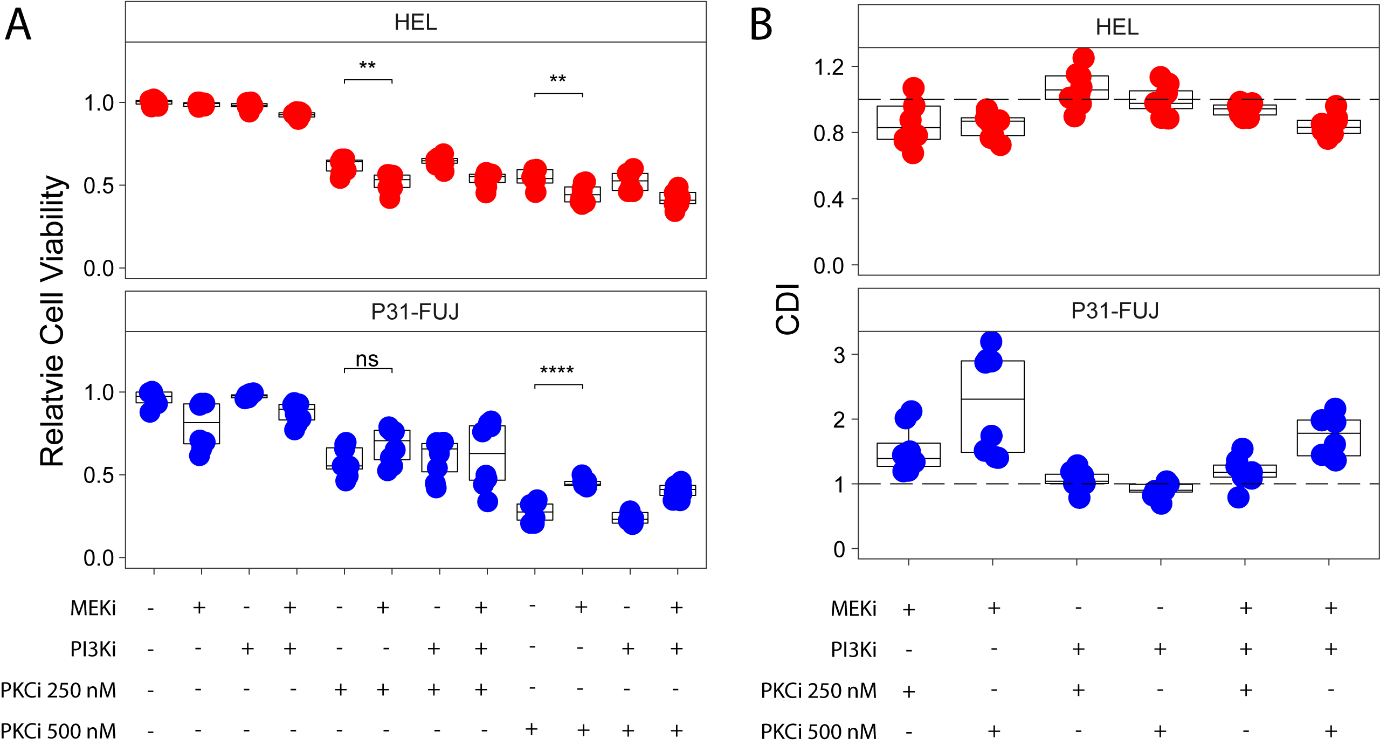


**Figure S8. Extent of PI3Ki, MEKi and PKCi synergism in P31-FUJ and HEL cells.**

(A) Response of HEL and P31-FUJ cells to the co-treatments with the named inhibitors. (B) Synergy quantification for co-treatments with the indicated inhibitors. CDI, coefficient of drug interaction; MEKi and PI3Ki were used at 1µM. MEKi, trametinib; PI3Ki, GDC-0941; PKCi, midostaurin.

RESOURCES TABLE

| **REAGENT or RESOURCE** | **SOURCE** | **IDENTIFIER** |
| --- | --- | --- |
| **Antibodies** | | |
| Rabbit polyclonal Phospho-AKT (S473) | Cell Signaling Technology | 9271 |
| Rabbit polyclonal Phospho-AKT (T308) | Cell Signaling Technology | 9275 |
| Rabbit polyclonal AKT | Cell Signaling Technology | 9272 |
| Rabbit monoclonal Phospho-PRAS40 (T246) | Cell Signaling Technology | 2997 |
| Mouse monoclonal Phospho-ERK1/2 (T202/Y204) | Cell Signaling Technology | 9106 |
| Rabbit monoclonal ERK1/2 | Cell Signaling Technology | 4695 |
| Rabbit polyclonal Phospho-p70S6K (T389) | Cell Signaling Technology | 9205 |
| Rabbit polyclonal Phospho-4EBP1 (T37/46) | Cell Signaling Technology | 9459 |
| Rabbit polyclonal Phospho-eEF2K (S366) | Cell Signaling Technology | 3691 |
| Rabbit polyclonal eEF2K | Cell Signaling Technology | 3692 |
| Rabbit polyclonal Phospho-eEF2 (T57) | Cell Signaling Technology | 2331 |
| Rabbit polyclonal eEF2 | Cell Signaling Technology | 2332 |
| Rabbit monoclonal β-Actin | Cell Signaling Technology | 8457 |
| Rabbit polyclonal GAPDH | Sigma-Aldrich | ab9485 |
| Rabbit monoclonal PI3K delta | Cell Signaling Technology | 34050 |
| Rabbit monoclonal p70S6K | Cell Signaling Technology | 2708 |
| Mouse monoclonal Puromycin | Merck Millipore | MABE343 |
| Anti-mouse IgG, HRP-linked | Cell Signaling Technology | 7076S |
| Anti-rabbit IgG, HRP-linked | GE Healthcare Life Sciences | NA934-1ML |
| **Chemicals, peptides, and recombinant proteins** | | |
| Trametinib (GSK1120212) | Selleckchem | S2673 |
| Pictilisib (GDC-0941) | Selleckchem | S1065 |
| A484954 | Tocris | 4483 |
| RPMI 1640 Medium | ThermoFisher Scientific | 61870010 |
| DMEM, high glucose | ThermoFisher Scientific | 31966021 |
| MEM alpha | ThermoFisher Scientific | 22571020 |
| Opti-MEM Reduced Serum Medium | ThermoFisher Scientific | 31985062 |
| Heat Inactivated Fetal Bovine Serum | ThermoFisher Scientific | 10500-064 |
| Penicillin-Streptomycin (10,000 U/mL) | ThermoFisher Scientific | 15140122 |
| Phosphate Buffered Saline (PBS) | Sigma-Aldrich | D8537 |
| Eppendorf Protein LoBind tubes, 1.5 mL | Sigma-Aldrich | Z666505-100EA |
| Eppendorf Protein LoBind tubes, 2 mL | Sigma-Aldrich | Z666513-100EA |
| Sodium Fluoride (NaF) | Sigma-Aldrich | 201154 |
| Sodium Orthovanadate (Na_3_VO_4_) | Sigma-Aldrich | 450243 |
| Sodium β-glycerophosphate | Sigma-Aldrich | G9422 |
| Sodium pyrophosphate (Na_4_P_2_O_7_) | Sigma-Aldrich | 71501 |
| Urea | Sigma-Aldrich | U5378 |
| HEPES sodium salt | Sigma-Aldrich | H7006 |
| DTT | ThermoFisher Scientific | R0862 |
| Iodoacetamide | Sigma-Aldrich | I1149 |
| Water for LC-MS (Optigrade) | LGC | SO-9368-B025 |
| Acetonitrile for LC-MS (Optigrade) | LGC | SO-9340-B025 |
| Trifluoroacetic acid UHPLC-MS (Optigrade) | LGC | SO-9668-B001 |
| Glycolic acid | Fisher Scientific | 10746561 |
| Ammonium acetate | Sigma-Aldrich | 09689 |
| Ammonia solution | LGC Standards | HPA-0070-B010 |
| Formic acid | ThermoFisher Scientific | F-1850-PB08 |
| Enolase | Waters | 186002325 |
| Guava ViaCount reagent | Merck Millipore | 4000-0040 |
| Lipofectamine 3000 Transfection Reagent | ThermoFisher Scientific | L3000015 |
| Protease inhibitor cocktail | Sigma-Aldrich | P8340 |
| PMSF | Sigma-Aldrich | 93482 |
| Okadaic acid | Sigma-Aldrich | O7885 |
| NuPAGE 4-12% Bis-Tris Protein Gels, 1.0 mm | ThermoFisher Scientific | NP0326BOX |
| NuPAGE MOPS SDS running buffer 20x | ThermoFisher Scientific | NP0001 |
| NuPAGE LDS sample buffer 4x | ThermoFisher Scientific | NP0007 |
| NuPAGE Transfer buffer 20x | ThermoFisher Scientific | NP0006 |
| NuPAGE Antioxidant | ThermoFisher Scientific | NP0005 |
| iBlot Transfer Stack, nitrocellulose | ThermoFisher Scientific | IB301001 |
| SuperSignal West Pico Plus | ThermoFisher Scientific | 34577 |
| Puromycin | Sigma-Aldrich | 540411 |
| Immobilized Trypsin, TPCK treated | ThermoFisher Scientific | 20230 |
| Oasis HLB 1cc cartridge, 10 mg | Waters | WAT094225 |
| Titansphere beads | GL Sciences | 5020-75010 |
| PE-filtered spin tips | Glygen | TF2EMT |
| **Critical Commercial Assays** | | |
| Pierce BCA protein assay kit | ThermoFisher Scientific | 23227 |
| **Experimental Models: Cell lines** | | |
| Human: MCF7 | ATCC | HTB-22 |
| Human: NTERA2 | ATCC | CRL-1973 |
| Human: HL60 | ATCC | CCL-240 |
| Human: CMK | DSMZ | ACC-392 |
| Human: HEL | DSMZ | ACC-11 |
| Human: KASUMI-1 | ATCC | CRL-2724 |
| Human: P31/FUJ | JCRB | 0091 |
| Human: THP-1 | ATCC | TIB-202 |
| Human: MV4-11 | ATCC | CRL-9591 |
| Human: MOLM13 | DSMZ | ACC-554 |
| Human: KMOE-2 | DSMZ | ACC-37 |
| Human: ML-2 | DSMZ | ACC-15 |
| Human: NOMO-1 | DSMZ | ACC-542 |
| Human: OCI-AML2 | DSMZ | ACC-99 |
| **Oligonucleotides** | | |
| ON-TARGETplus SMART pool against human *eEF2K* siRNA | Dharmacon | L-004950-00-0005 |
| ON-TARGETplus Non-targeting Control pool | Dharmacon | D-001810-10-05 |
| **Software and Algorithms** | | |
| Xcalibur | ThermoFisher Scientific | https://www.thermofisher.com/search/results?query=xcalibur%E2%84%A2&navId=12141&persona=Catalog |
| Mascot Daemon (2.5.0) | Matrix Science | http://www.matrixscience.com/ |
| Mascot Distiller (v2.5.1.0) | Matrix Science | http://www.matrixscience.com/ |
| Mascot Search engine (v2.5) | Matrix Science | http://www.matrixscience.com/ |
| Pescal | In-house | https://www.mcponline.org/content/6/9/1560.long |
| Gephi (v0.9.1) | Gephi | <https://gephi.org/> |
| CytoSoft (v2.5.7) | Luminex | <https://www.luminexcorp.com/guava-easycyte-software/> |
| Image Studio Lite (v5.2) | LI-COR | <https://www.licor.com/bio/image-studio-lite/download> |
| RStudio Desktop (v1.2.5033) | RStudio | https://rstudio.com/ |
| ggplot2 package (v3.3.1) | CRAN r project | <https://cran.r-project.org/web/packages/ggplot2/index.html> |
| Prism 8 | GraphPad | <https://www.graphpad.com/scientific-software/prism/> |
| Adobe Illustrator | Adobe | <https://www.adobe.com/uk/products/illustrator.html> |
| **Other** | | |
| EASY-Spray source | ThermoFisher Scientific | ES081 |
| µ-pre-column Acclaim PepMap 100 C18 LC | ThermoFisher Scientific | 160454 |
| Analytical column Acclaim PepMap 100 C18 LC | ThermoFisher Scientific | 164569 |
| **Deposited data** | | |
| Mass spectrometry phosphoproteomics for 3 cell lines | This study | PRIDE: PXD018873 |
| Proteomics and phosphoproteomics data from 12 AML cell lines | (14) | PRIDE: PXD019591 |
| Original data from Western blots | This study | Mendeley Data http://dx.doi.org/10.17632/xjsdxnpxkb.1 |
